# Hippocampal tangential insertions of high-density silicon probes in head- fixed mice enhance spatial sampling

**DOI:** 10.1016/j.isci.2026.118131

**Published:** 2026-11-05

**Authors:** Annu Kala, Vera Evander, John J. Tukker, Jens Kremkow, Dietmar Schmitz, Jérémie Sibille

**Affiliations:** 1 Charité-Universitätsmedizin Berlin, Corporate Member of Freie Universität Berlin and Humboldt-Universität Berlin, Institute of Cell Biology and Neurobiology, 10117 Berlin, Germany; 2 Charité-Universitätsmedizin Berlin, Corporate Member of Freie Universität Berlin and Humboldt-Universität zu Berlin, Neuroscience Research Center, 10117 Berlin, Germany; 3 German Center for Neurodegenerative Diseases (DZNE) Berlin, 10117 Berlin, Germany; 4 Charité-Universitätsmedizin Berlin, Corporate Member of Freie Universität Berlin and Humboldt-Universität Berlin, Einstein Center for Neuroscience, 10117 Berlin, Germany; 5 Charité-Universitätsmedizin Berlin, Corporate Member of Freie Universität Berlin and Humboldt-Universität Berlin, NeuroCure Cluster of Excellence, 10117 Berlin, Germany; 6 Charité-Universitätsmedizin Berlin, Corporate Member of Freie Universität Berlin and Humboldt-Universität Berlin, Bernstein Center for Computational Neuroscience, Humboldt-Universität zu Berlin, 10117 Berlin, Germany; 7 Institute of Biology, Otto-von-Guericke-University Magdeburg, 39120 Magdeburg, Germany

**Keywords:** neuropixels, hippocampus, ripple spatio-temporal structure, functional connectivity, tangential insertion

## Abstract

The hippocampus is a key structure within the medial temporal lobe, which plays a central role in the formation and consolidation of declarative memories. Due to its complex croissant-like morphology, it has been difficult to use linear high-density probes to perform extracellular recordings from large continuous segments of the hippocampus. We here describe how to apply a tangential insertion approach to enable continuous recordings of the hippocampus. We propose two different insertions aligned to either the dorsal or ventral sections of the hippocampus. Both insertions are performed in head-fixed conditions, which can be easily combined with detailed monitoring of the animals’ behavioral states. We show that when placed across the pyramidal layer, they can harness ripple oscillations at an unprecedented spatial resolution, which also enabled us to record up to 400 hippocampal neurons simultaneously from a single probe, endowing us with the capability to capture putative functionally connected neuronal pairs.

## Highlights

• Tangential insertions can harness fine-scale spatiotemporal ripple’s organization
• A single tangential insertion yields up to 400 simultaneous hippocampal neurons
• Tangential insertions detect up to 243 parallel functional synaptic connections

## Graphical abstract

**Figure:**
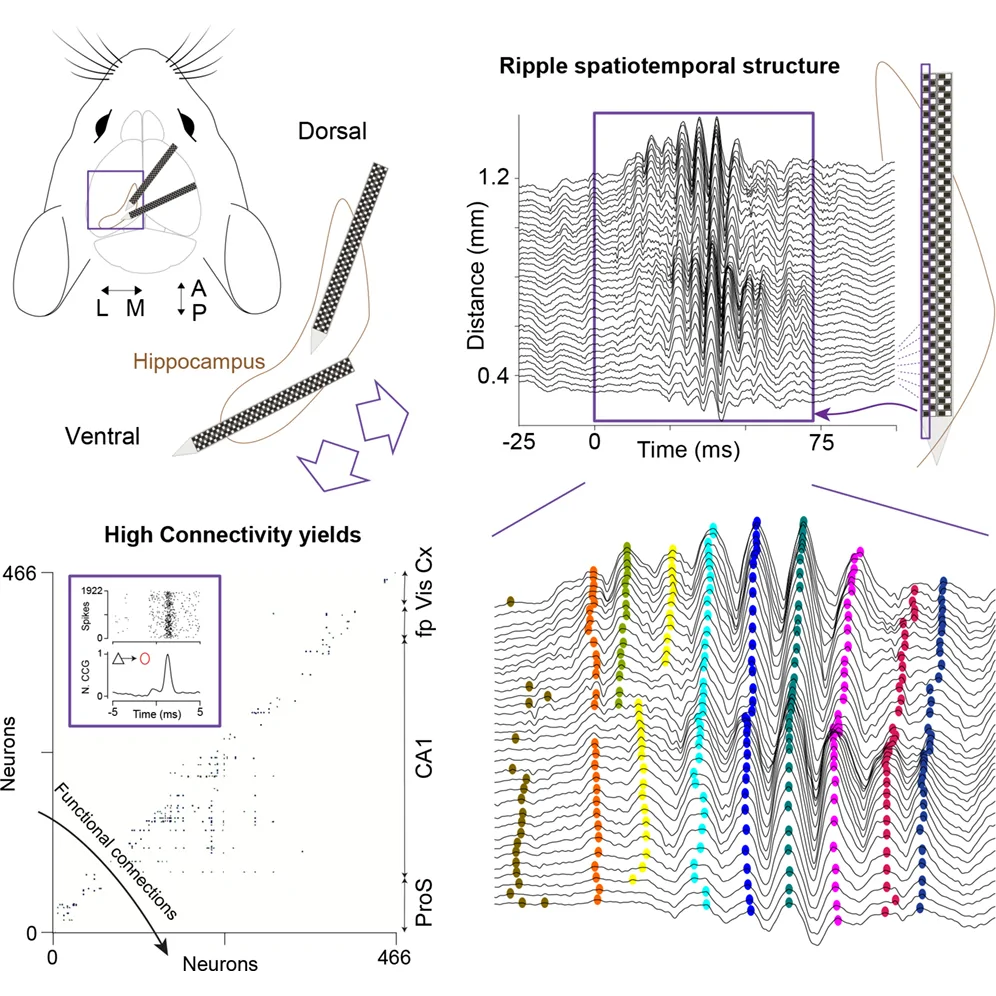

## Teaser

Neuroscience; Behavioral neuroscience; Techniques in neuroscience; Bioengineering; Biological sciences research methodologies

## Introduction

The hippocampus is one of the most studied regions in the mammalian brain. In fact, the very first “unit” recordings were made with glass microelectrodes from hippocampal pyramidal cells in the cat.^1^ This was expanded to single-cell recordings from awake behaving rats, revealing the existence of place cells encoding the spatial location of the animal.^2^ The introduction of techniques combining several wire electrodes^3^^,^^4^^,^^5^ or multiple recording points arranged in an array^6^^,^^7^ greatly expanded our knowledge of neural coding by enabling the recording of spatially distributed ensembles of cells. The most recent step in this development has been the introduction of high-density recording probes such as the Neuropixels probe (NP)^8^^,^^9^^,^^10^ with a denser spatial coverage enabling the recording of hundreds of single-units simultaneously. Higher numbers of recorded single-units not only enable the identification of relatively rare subpopulations but also maximize the identification of monosynaptically connected pairs of neurons^11^^,^^12^^,^^13^^,^^14^^,^^15^^,^^16^ and high-dimensional descriptions of neural population code.^17^^,^^18^^,^^19^ For such recordings, the spatial layout of recording sites is of crucial importance, not only to guarantee the largest possible number of recorded neurons, but also to reveal possible spatial structure in their activity. In the hippocampus, as in other cortical tissues, such structure may be found in a laminar or sublaminar organization, which has been studied extensively using vertical insertions, and also across subregions,^20^^,^^21^^,^^22^^,^^23^^,^^24^^,^^25^^,^^26^^,^^27^^,^^28^ where differences have been shown in connectivity and molecular expression profiles.^29^^,^^30^^,^^31^^,^^32^^,^^33^^,^^34^

Similarly, local field potential (LFP) recordings in the hippocampus have been studied for a long time,^35^ and oscillatory activity in various frequency ranges has been closely implicated in hippocampal function.^36^^,^^37^^,^^38^ Studies using vertical approaches showed that these oscillations are organized in a laminar manner, with specific “depth profiles.”^39^^,^^40^^,^^41^^,^^42^^,^^43^^,^^44^^,^^45^^,^^46^^,^^47^^,^^48^^,^^49^ In addition, the use of multiple vertical probes in different locations of the hippocampus has revealed propagation patterns of theta waves (∼*6–12 Hz*^50^^,^^51^^,^^52^)) and sharp-wave associated ripples (SWRs; ∼*130–250 Hz*) within and across subregions. Although these approaches have produced an incredible amount of data and insights, they do have a limited spatial resolution in terms of number of recording sites in “non-vertical” orientations, particularly along the antero-posterior axis of the hippocampus.

Finally, recording the brain activity from rodents in head-fixed conditions has supported a wealth of discoveries over the last two decades.^9^^,^^10^^,^^53^^,^^54^^,^^55^^,^^56^ The correspondence between brain activity in freely moving and head-fixed running animals is still debated^57^; nevertheless, accumulated evidence suggests that in head-fixed recordings (combined with a virtual reality system) both the visual cortex^55^ and the hippocampus^53^ exhibit visual and spatial modulations. Furthermore, head-fixed conditions permit precise control of different types of sensory cues^58^ and even enable single-cell patch-clamp in the hippocampus,^53^^,^^59^ which revealed a new form of behaviorally driven plasticity.^60^ Although head-fixation limits the study of certain aspects of behavior,^61^^,^^62^ it allows the characterization of more precise movement-related brain activity along with highly complex multi-probe insertion(s) at intricate angle(s).

Here, we present an alternative to vertical insertions of multiple wires or probe shanks. By inserting a single NP probe tangentially along the antero-posterior axis (Figure 1, in either the ventral or dorsal parts of the hippocampus), we can substantially increase the number of channels (Figure 1) within the hippocampus. This not only lets us map the distribution of LFP patterns at a high resolution across a large spatial extent (Figure 2) but also allows us to capture units (Figure 3) from an extended spatial domain across the pyramidal layer(s). Applying this approach in head-fixed recordings with high-resolution behavioral tracking makes it possible to correlate hippocampal activity with facial movements (Figure 4). Furthermore, large numbers of functional connections can also be accessed along the longitudinal axis (Figure 5). Overall, our approach enables the study of behavioral correlations with neuronal activity along the longitudinal axis of the hippocampus with higher precision.

**Figure 1:**
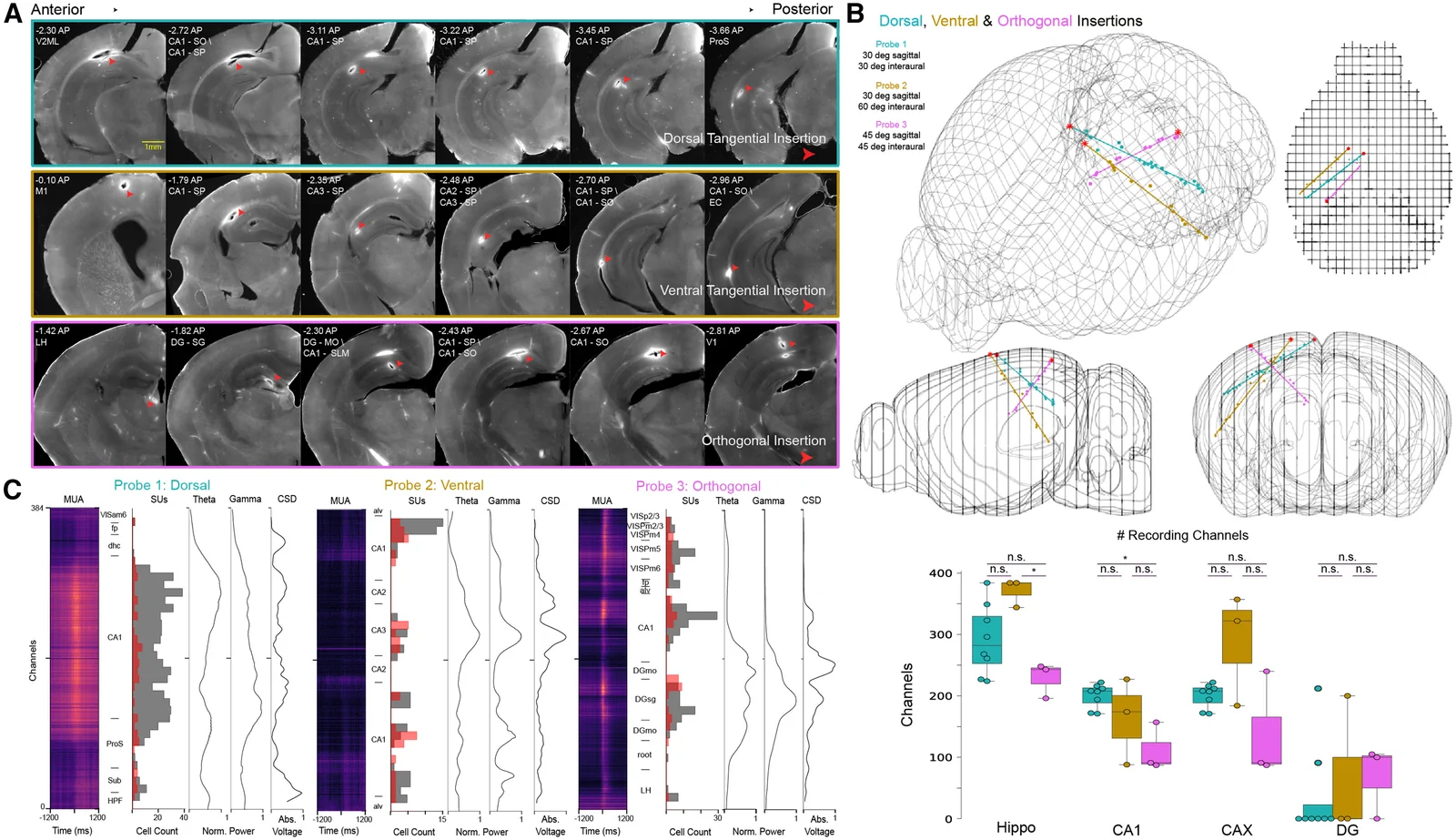
Hippocampal dorsal and ventral tangential insertions (A) Coronal sections from 3 different mice, illustrating the dorsal (top) and ventral (middle) tangential insertions, as well as a more standard orthogonal insertion (bottom), ordered in anterior (left) to posterior (right) planes. For each image, the AP distance relative to Bregma is indicated, along with the anatomical areas in which the probe track (red arrows) is located in this image. Colored arrows at the bottom of each row indicate the direction of each insertion. (B) 3D reconstructions of the positioning of the probes in the left-brain hemisphere showing the probe placement for the three insertions (red dots indicate insertion points at the brain surface). 3D perspective (top-left), with corresponding sagittal (bottom-left), coronal (bottom-right), and interaural (top-right) projections. Below is the quantification of the number of recording channels in the hippocampus, CA1, general cornu ammonis regions (CAX), and DG by insertion (boxplot, bottom right). (C) Illustration of the physiological alignment in a few recordings: for the dorsal (left), ventral (middle), and orthogonal (right) insertions also shown in (B). For each of the *3* probes, for all recorded channels, plots show the mean normalized firing rate of multi-unit activity (MUA) aligned to movement onset (left), the number of isolated single-units (SUs/10 channels) obtained after spike sorting and curation (second from left; narrow-waveform units, red; broad-waveform units, gray), the LFP power in the theta (third from left; *4–10 Hz*) and gamma (fourth from left; *30–100 Hz*) bands, together with the channels’ anatomical locations deduced from the 3D reconstructions (between MUA and SU density). Plots also show the absolute value of the CSD along the probe when time-locked to theta trough (right) (cf. Figure S2). The list of anatomical abbreviations can be found in Table 1. Data is represented as mean ± SEM.

**Figure 2:**
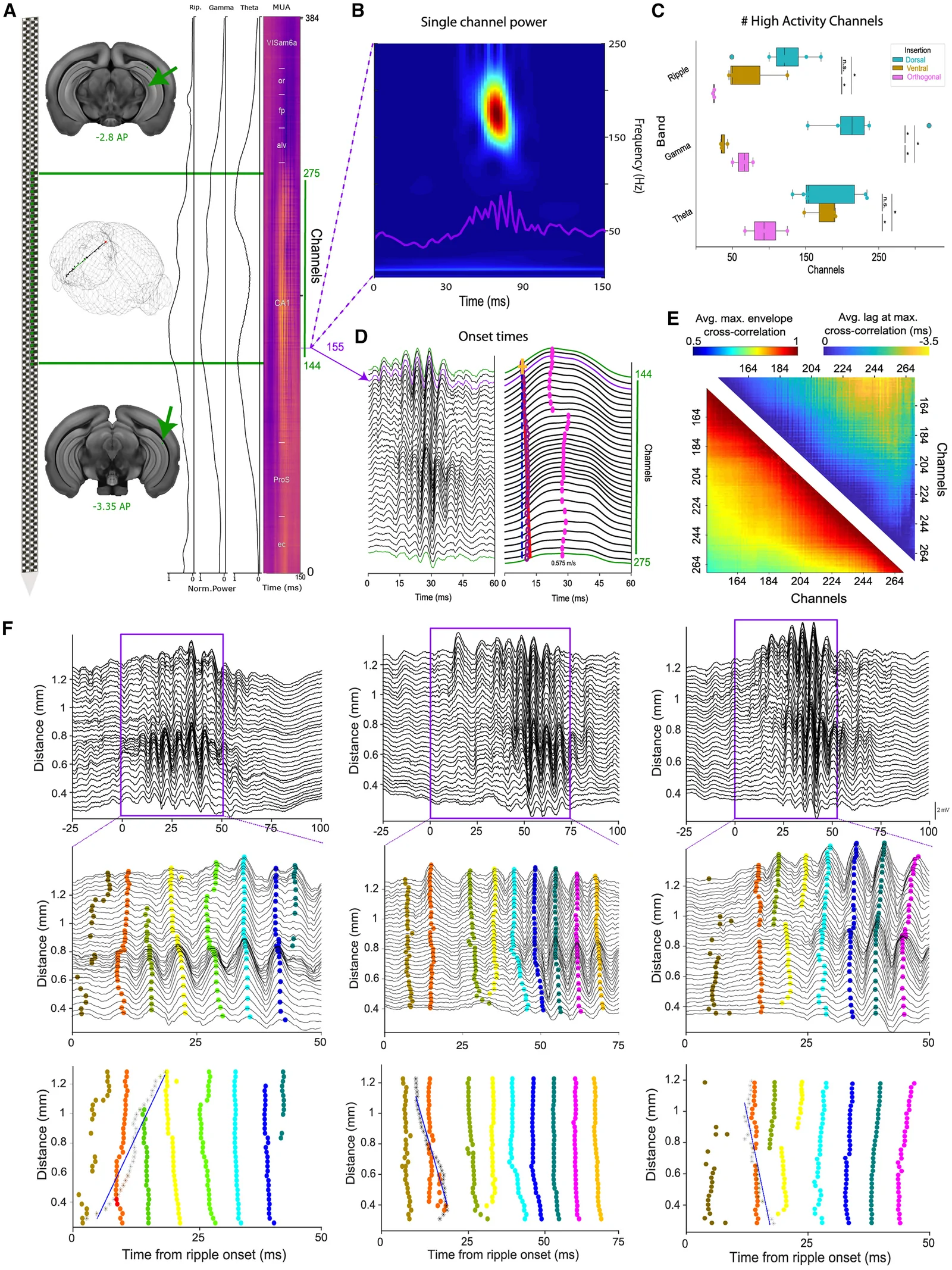
Bidirectional awake ripple propagation (A) Reconstructions of the example probe used throughout this figure. Green channels indicate the channels located close to the pyramidal layer shown in (C)-(H) (left). Coronal slices and the green shaded section show the *1.29 mm* region of dorsal CA1 along which individual ripples are tracked (Middle). LFP power profile in the theta (*4–10 Hz*), gamma (*30–100 Hz*), and ripple (*110–240 Hz*) ranges, as in Figure 1 (Right). Multi-unit activity in each channel overlaid with anatomical locations of the probe, aligned to the ripple event shown in (C) and (E). See Table 1 for the list of abbreviations. (B) Stockwell-transformed power spectrum of channel *155* centered on one ripple event. White trace is the corresponding LFP signal from this channel. (C) Number of channels that have a normalized power greater than *0.5* for the theta and gamma frequency bands as indicated, with recordings aligned to movement onset as in Figure 1C. Ripple quantification (top) indicates the number of channels along which ripple activity could be detected. (D) Raw LFP traces for an example ripple event (Left). Every four channels (green channels in A) are shown (Right). Corresponding envelope of the Hilbert-transformed ripple-range-filtered signal for each channel. Yellow crosses indicate the channel on which the ripple was first detected. Purple circles indicate the time of crossing the 50% highest amplitude of the envelope. The red line is the linear polynomial fit across these time points measuring the different activation time, indirectly showing the propagation of ripples. Dashed blue lines represent zero lag w.r.t the yellow crosses. Average speed across the channels is calculated as (channels traveled⁎inter-channel-spacing)/ (delay to *50%* threshold crossing). Pink circles indicate the time of peak ripple power per channel. (E) Heatmaps showing the average max-envelope cross correlation (bottom) and the corresponding lag (top) between all channels within our ripple-area (channels *144–275*, *n* = *105* ripple events). (F) Channel-resolved ripple dynamic in three representative ripples (left, middle, and right). The uppermost row shows raw LFP signal illustrating ripple activity across extended spatial domains. Middle row shows peaks (colored circles) highlighting cycle-by-cycle variations during ripples. Third row represents ripple band Hilbert envelope across channels with black markers indicating the time of crossing the 50% highest amplitude of the envelope, blue lines indicating the linear fits of the crossings, and colored dots indicating the peak times from the plots in the middle row. Note the spatial pattern of ripple peaks does not simply follow the propagation as defined by the power in the ripple band (black markers, blue line). Data is represented as mean ± SEM.

**Figure 3:**
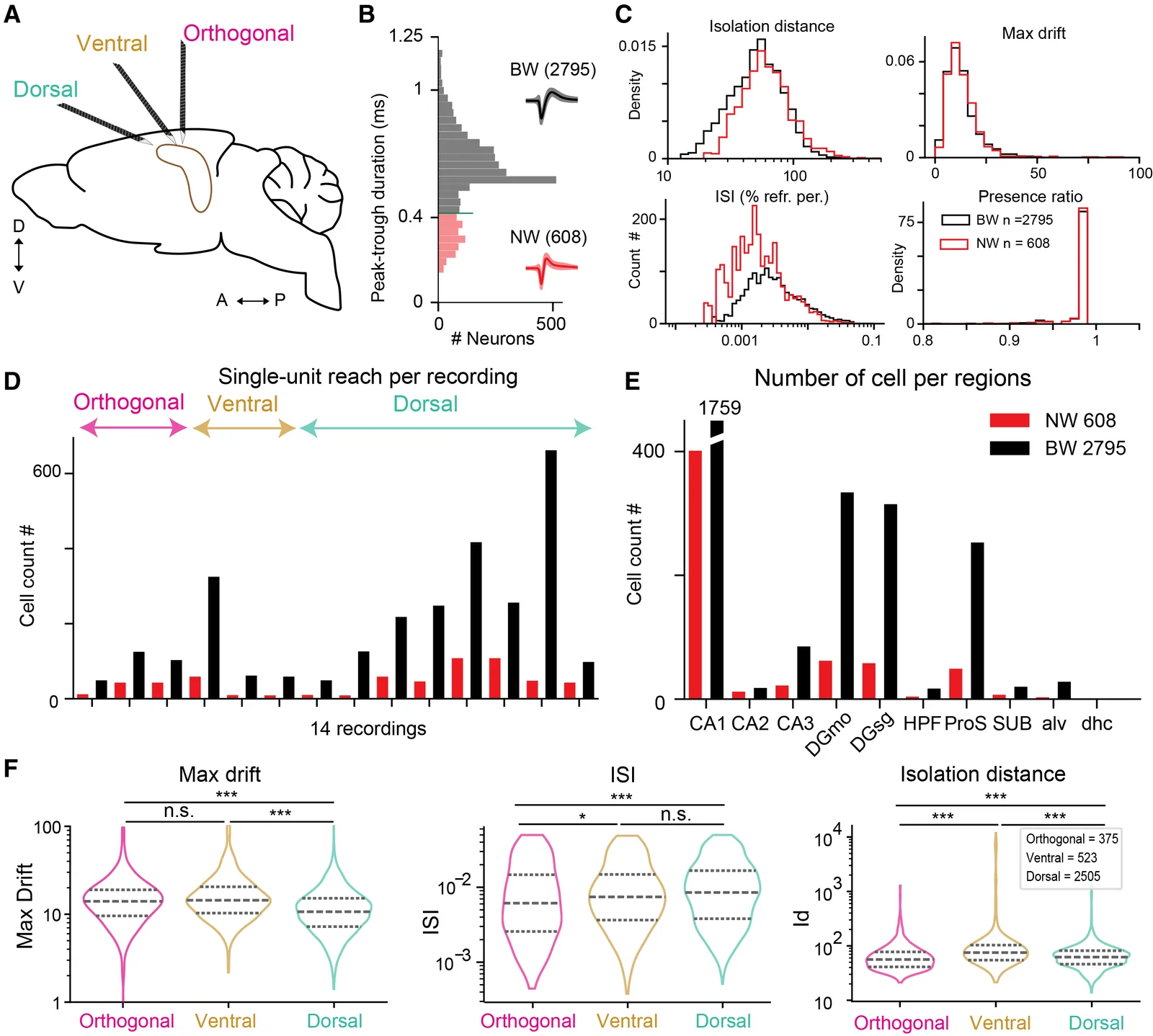
Single-unit reach, quality metrics (A) Illustration of the three insertion types. (B) Quantification of the peak-trough duration illustrating the difference between broad and narrow waveforms (BW vs. NW), in the hippocampal formation. (C) Quality metrics of NW and BW neurons showing their high quality in isolation distance, inter-spike interval (ISI), and presence ratio, together with the higher stability (max drift) gained from the tangential insertion (*n* = *14* recordings from *14* mice). (D) Quantification of NW and BW cell numbers for each insertion type. (E) Quantification of NW and BW neuron numbers in each region identified from the 3D reconstruction (summed over all recordings). Note that the seemingly paradoxical higher number of neurons obtained in DG-mo compared to DG-sg may be related to the tangential positioning of the probe above the DG, thereby overestimating the number of channels in the DG-mo when the spikes may stem from the DG-sg. (F) Comparative quality metrics obtained in the different types of insertions, max drift (left), ISI, for refractory period violation (middle), and isolation distance (right). (*n* = *375/523/2,505* neurons from n mice = *3/3/8* for orthogonal, ventral, and dorsal insertions respectively; Wilcoxon rank sum tests). Data are represented as mean ± SEM.

**Figure 4:**
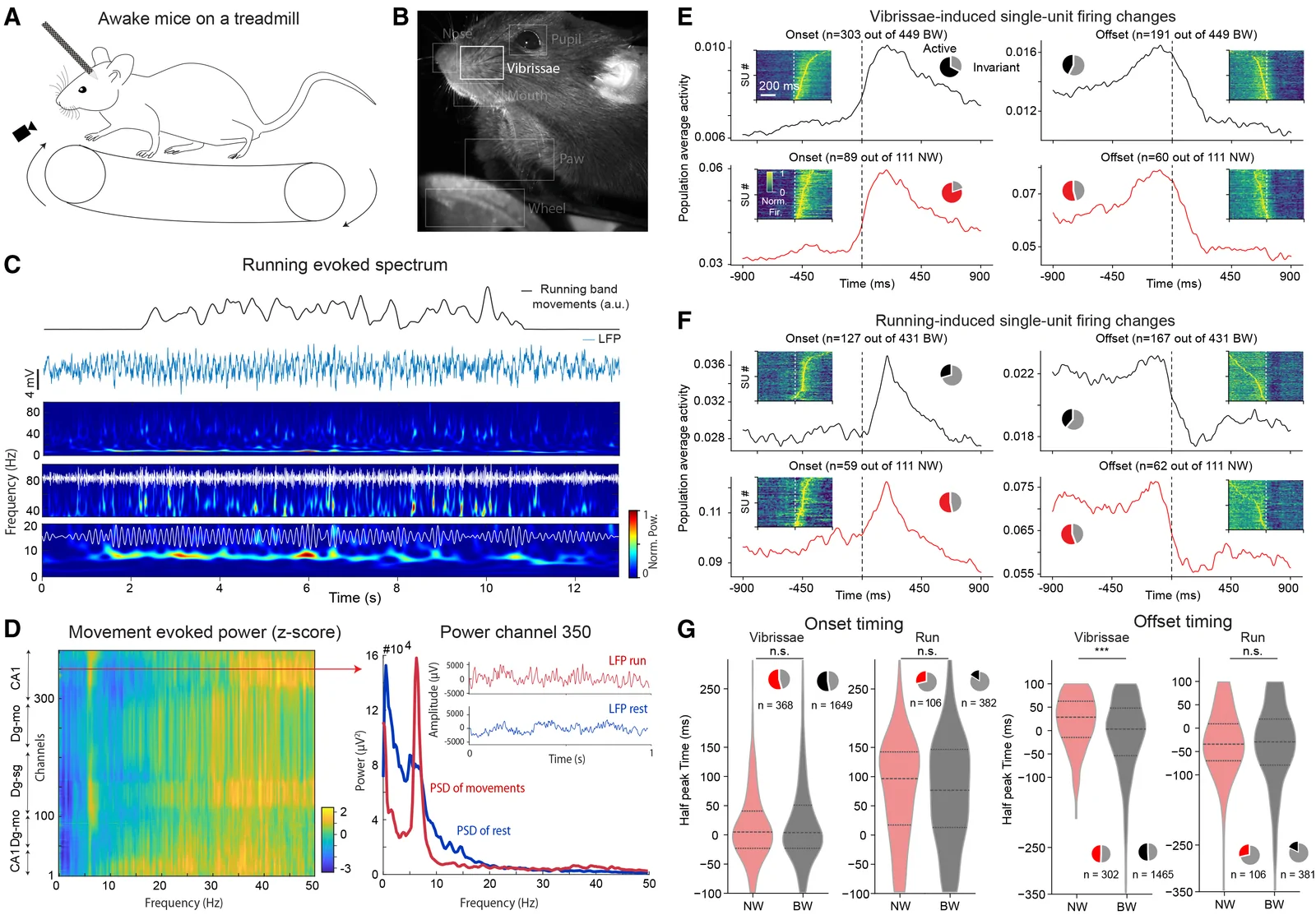
Coactivation of hippocampus with running and facial movement (A) Schematic of recording set-up: the acute head-fixed recording is combined with facial monitoring and quantification of the running behavior based on the analog signal measured from the running band. (B) Illustration of the facial video, to show the region of interest used to characterize vibrissae movements (other regions labeled in light gray can be tracked; Figure S7). Facemap was used to extract the singular value decomposition (SVD) to isolate moments of heightened activity versus rest (Figure S7). (C) Representative *13* s of the normalized running traces from the running band, in arbitrary units (Top). Corresponding LFP (Bottom). Time-frequency representation from the LFP using Stockwell transformation (Top spectrogram). Zoomed high frequencies (*40–90 Hz*, Middle spectrogram). Zoomed low frequencies (*0–20 Hz*, Bottom spectrogram). The last two spectrograms also show corresponding bandpass-filtered LFP signal overlaid in white. (D) Log-normalized relative increase in power in each channel for movement periods vs. silent periods (left). Blue and red lines show the corresponding PSD of movement and silent epochs on channel *350* (right). (E) Mean population increase in firing of BW (top) and NW (bottom) neurons relative to the onset (left) and offset (right) of whisking moments (t = *0*) within a single recording. Insets illustrate the normalized firing changes of all neurons showing a meaningful change of activity during onset and offset of facial movement. Pie plot insets illustrate the proportion of modulated (Active) versus invariant (Invariant) neurons during whisking (*n* = *1* mouse). (F) Equivalent measures in the same recording of BW and NW neurons to the onset (left) and offset (right) of running bouts (*n* = *1* mouse). (G) Dataset estimates of half-peak delay times of the hippocampal neuronal responses relative to the onset (left) and offset (right) of vibrissae and run movements. Violin plots show all the half-peak times to illustrate the spread of the obtained values: the middle-dashed lines show the mean values; top and bottom lines show the *25*^*th*^ and *75*^*th*^ quartile. Pie plots show proportions of active (Active) versus invariant (Invariant) neurons throughout the dataset (*n* = 8 mice for running behavior, *n* = 14 mice for vibrissae movements; all statistics are done using the Wilcoxon rank-sum test). Data is represented as mean ± SEM.

**Figure 5:**
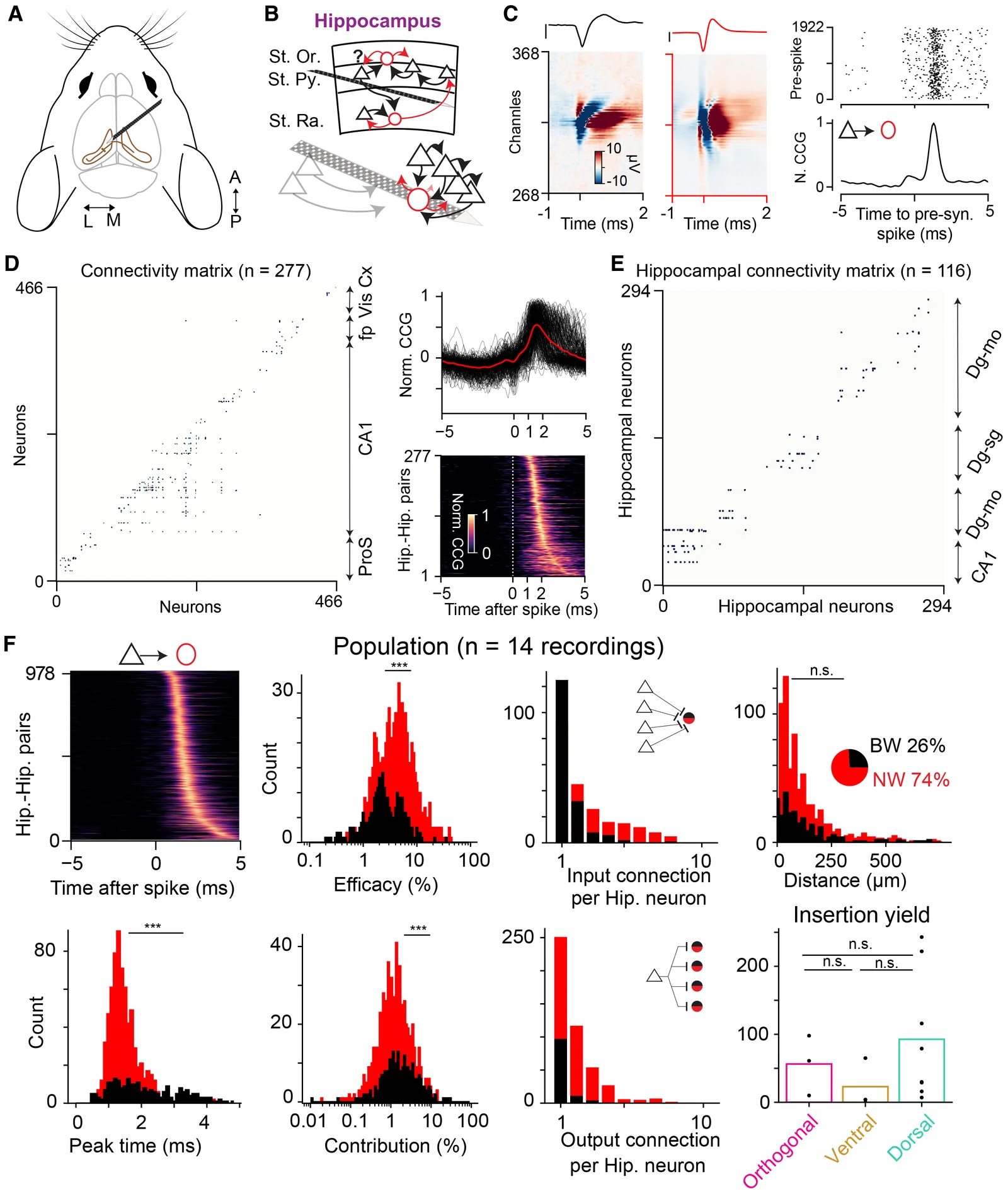
Functional excitatory connectivity within the hippocampus (A) Schematic of the NP placement for a tangential dorsal insertion. (B) Schematic connectivity measure within the hippocampus between BW neurons and other neuron(s): other BW (black) or NW (red) neurons. (C) Illustration of a single excitatory synaptic contact of a BW neuron toward an NW neuron. Each neuron is shown with its single-channel waveform (top, scale bar is *50 μV*) and below its 2D multi-channel waveform, illustrating its different spatial and temporal extent. Functional excitatory contacts are identified by their typical prominent peak at a monosynaptic time delay between the pre- and post-synaptic spiking activity (cf. raster plot and corresponding CCG, bottom right). (D) Typical connectivity matrix in a single recording showing *277* excitatory connections between the available *466* neurons (neurons are organized based on their increasing channel position on the probe, left), with their corresponding reconstructed brain regions (double arrow middle). The corresponding normalized CCG profiles (top right; *n = 277; 243* pairs are in the hippocampal formation) and the corresponding heatmap plotted signals (bottom right). (E) Another connectivity matrix example, here illustrating more regional connections in this other recording. (F) Summary and quantification of all available connections within the dataset (*n* = *980* pairs, from *14* recordings, n = *0–270* pairs per recording). A large majority of detected excitatory monosynaptic connections are toward NW neurons, as illustrated in the pie chart inset (*22%* of connections are BW to BW neurons). Quantifications of (top left to right): CCG profiles, efficacy, convergence, and distance between the connected neuronal pairs; (bottom left to right): peak times after spikes (with the mean population values), contribution, and divergence connectivity scheme. The last bottom right panel illustrates the differences of synaptic contact reach between orthogonal, tangential ventral, and dorsal insertion (*n* mice *= 3/3/8*). All statistics are done using the Wilcoxon rank-sum test). Data is represented as mean ± SEM.

## Results

### Tangential insertions maximize spatial sampling yields in the hippocampus

First, to illustrate the advantages of tangential hippocampal insertions, we compared the localization of channels within the hippocampal formation across different insertion types (Figure 1B, Table 1 for abbreviations). Although qualitatively we observed more channels in the hippocampus as a whole, in CA1, and in CA1-3 (which we denote as CAX) in the tangential insertions than in the more orthogonal ones, the difference was only statistically significant for the number of channels across the whole hippocampus in tangential ventral (*371 ± 23, n = 3*) versus orthogonal insertions (*229 ± 29, n = 3*) (*p* = *0.049*, Wilcoxon rank-sum), and for the number of CA1 channels in tangential dorsal (*200 ± 19, n = 8*) versus orthogonal insertions (*112 ± 39, n = 3*) (*p* = *0.014*, Wilcoxon rank-sum). Given the very small number of orthogonal insertions (*n* = 3) in our dataset, such comparisons are statistically under-powered, but the trend for higher number of hippocampal channels in the tangential insertions is clear (also in comparison to other datasets from more purely orthogonal recordings, see discussion), except for DG.

**Table 1:** List of anatomical abbreviations

alv	Alveus of the hippocampus	LH	Lateral habenula
CA1/2/3	Cornu ammonis 1/2/3	MOs2/3	Secondary motor area, layer 2/3
ccb	Corpus callosum, body	or	Optic radiation
ccs	Corpus callosum, splenium	ProS	Pro-subiculum
cing	Cingulum bundle	root	root
DG-mo	Dentate gyrus, molecular layer	RSPagl5,6	Retrosplenial area, lateral agranular part, layer 5,6
DG-po	Dentate gyrus, polymorph layer	scwm	Supra-callosal cerebral white matter
DG-sg	Dentate gyrus, granule cell layer	SSP-tr6	Primary somatosensory area, trunk, layer 6
dhc	Dorsal hippocampal commissure	Sub	Subiculum
ec	External capsule	TEa5,6	Temporal association areas, layer 5,6
Ent-6a	Entorhinal area, lateral part, layer 6a	VISp2/3,5,6	Primary visual area, layer 2/3,5,6
fp	Corpus callosum posterior forceps	VISam6	Anteromedial visual cortex, layer 6
HPF	Hippocampal formation	VISpm2/3,5,6	Posteromedial visual cortex, layert 2/3,4,5,6

Second, we characterized the LFP profiles across all the channels, comparing the number of channels with supra-threshold ripple, gamma, and theta band activity across each insertion type (Figure 2B). Despite the low statistical power mentioned above, the number of channels across which we could detect ripples (Figure 2B, top) was significantly larger during tangential compared with orthogonal insertions (dorsal (*120 ± 39, n = 7*) vs. orthogonal (*24 ± 1, n = 3): p = 0.017*); ventral (*73 ± 45, n = 3*) vs. orthogonal: *p* = *0.049*, Wilcoxon rank-sum; note one dorsal recording had to be excluded due to lack of ripples). Because of our much higher spatial sampling within the hippocampus, we can track individual ripples across large swaths of the hippocampus using this method (Figure 2). We also found a broader coverage of oscillatory gamma and theta activity in tangential insertions compared to orthogonal insertions. The number of channels encompassing higher theta power was significantly enhanced in both kinds of tangential insertions (dorsal (*177 ± 42, n = 8*) vs. orthogonal (*95 ± 29, n = 3*): *p* = *0.014*; ventral (*176 ± 24, n = 3*) vs. orthogonal: *p = 0.049*, Wilcoxon rank-sum), while the number of channels encompassing higher gamma power was enhanced for dorsal tangential insertions (dorsal (*220 ± 48, n = 8*) vs. orthogonal (*65* ± *14, n = 3*): *p* = *0.014*) and decreased for ventral tangential insertions (ventral (*37 ± 5, n = 3*) vs. orthogonal: *p* = *0.049*, Wilcoxon rank-sum).

To better illustrate the added value of the higher sampling offered by tangential insertions, we characterized the spatiotemporal dynamics of ripples at unprecedented resolution by comparing the timing of different points within each ripple event (Figure 2). Specifically, comparing in each channel the ripple onset (from the Hilbert transform, cf. methods) against the ripple peak showed discontinuities in the ripple peak timing shift, which were not apparent when tracking the onset (Figure 2E). Tracking each cycle peak, we observe a more complex spatiotemporal structure (Figure 2F). Thus, the high spatial resolution of our method reveals that ripple propagation may not be smooth across the hippocampal network: along the antero-posterior axis of the hippocampus, ripples can exhibit propagation jumps, and cycle-specific propagation dynamics, which differ from one ripple to the next. Furthermore, we analyzed the spatiotemporal organization of ripple frequencies and found clear positive/negative gradients indicating diverse directionality (Figures S5A–S5C). Comparison between frequency-based directionality and ripple band envelope-based directionality broadly shows limited overlap suggesting different features of ripple dynamics (Figure S5D).

### Tangential insertions maximize cell and functional connectivity yields in the hippocampus

Third, we investigated hippocampal single-unit yield across all insertion types. Maximal cell yield optimized in a single recording in dorsal tangential insertion reached about *710* units compared to *384* and *168* units (Table 2) in ventral tangential and orthogonal insertions respectively. Qualitatively, it seems that the yields are highest in the dorsal tangential insertions, but again the low statistical power limits the certainty of such comparisons: we did not observe any group-level significance (Figure 3D) in the cell reach across different insertion types (dorsal (*313 ± 218, n = 8*)/ventral (*174 ± 182, n = 3*): *p* = *0.497*; dorsal/orthogonal (*125 ± 57, n = 3*): *p* = *0.376*; ventral/orthogonal: *p* = *1.000*, Wilcoxon rank-sum).

**Table 2:** Table summarizing recording wise the number of channels in the hippocampus

Mouse	Insertion	Ch. in hip.	Ch. in CA1	Cell yield	Syn. yield	Theta (ch)	Gamma	Ripple
12	Orthogonal	243	91	61	10	67	78	25
13	Orthogonal	196	87	168	98	125	66	23
14	Orthogonal	248	157	146	61	93	50	25
9	Ventral	384	88	384	65	148	43	45
10	Ventral	384	227	71	5	99	73	125
11	Ventral	344	174	68	29	191	35	50
7	Dorsal	261	222	59	0	156	201	100
8	Dorsal	224	171	135	4	152	237	120
1	Dorsal	296	216	277	79	132	319	49
2	Dorsal	384	172	294	116	232	153	171
3	Dorsal	323	212	525	222	234	228	no ripple
4	Dorsal	268	194	364	243	212	194	129
5	Dorsal	349	207	710	30	147	199	151
6	Dorsal	227	208	141	16	152	226	121

In CA1, the number of cell yield, of synaptic yield, and the number of channels with enhanced power in theta, in gamma range, and with ripple activity.

Fourth, we compared several measures of recording quality between different insertion types. Similar to what has been described for tangential insertions in the superior colliculus,^63^ we report a significantly lower drift (Figure 3F left panel) in dorsal hippocampal tangential insertions (*12.01 ± 7 μm, n = 8*) compared to orthogonal (*15.75 ± 10.8 μm, n = 3; p = 3.49 × 10⁻¹⁶*; Wilcoxon rank-sum test) or ventral tangential insertions (*15.35 ± 11 μm, n = 3; p = 1.08 × 10⁻¹⁴*; Wilcoxon rank-sum test). Inter-spike interval (ISI) and isolation distance were also significantly improved in tangential insertions compared to orthogonal (Figure 3F middle and right panels; comparisons for dorsal vs. ventral, dorsal vs. orthogonal, and ventral vs. orthogonal: ISI, *p* = *0.28/0.006/0.03*; isolation distance, *p* = *4.07 × 10⁻⁹/0.001/4.36 × 10⁻⁶*; Wilcoxon rank-sum tests, *n* = *2,505/523/375* neurons from n mice = *8/3/3* for dorsal, ventral, and orthogonal, respectively).

We next investigated the possibility to combine our insertions with behavioral tracking as classically done with head-fixed awake experimentation. We found a profound temporal relationship between neural activity and whisking compared to running bouts for both onset and offset of movements (Figure 4G). We note that the larger delays in onset and offset responses to running bouts versus whisking may just reflect the delays that exist between the bodily movement in the mouse and the actual band movement during run. This delay may also partially underlie the earlier emergence of theta oscillations in the hippocampal LFP compared to the running band signal (Figure 4C). Taking the paw/breast motions into account (which can be tracked from the facial video, Figure 4B) could potentially resolve this issue but is unfortunately confounded by other movements such as respiration (cf. Figure S7).

The kinetics of recruitment in the beginning and end of whisking bouts (onset & offset, for Facemap and run) showed very small differences in broad waveform (BW) versus narrow waveform (NW, cf. methods) neurons for vibrissae movements (av. onset *= 22.9 ± 79/15.1 ± 63* ms for BW and NW respectively; *p* = *0.5*, *n* = *1,649/368* units, *n* = *14* mice; av. offset = −*16 ± 93/16 ± 59* ms for BW and NW respectively; *p* = *2.51 × 10⁻⁸*, *n* = *1,465/302* units, *n* = 14 mice, Wilcoxon rank-sum). During a run bout, in agreement with the delay observed on theta oscillations, we report similar delay at the level of single-units (av. onset = *76 ± 98/79 ± 92* ms for BW and NW respectively.; *p* = 0.63; av. offset = −40 ± *94/−39 ± 92* ms for BW and NW respectively, *p* = *0.88*, *n* = *106/382* units, *n* mice = *8*, Wilcoxon rank-sum).

Fifth, we compared the functional connectivity by quantifying the excitatory synaptic contacts across all insertion types and found no statistical significance (Figure 5F, average pairs detected = *88 ± 97/33 ± 29/56 ± 44*, dorsal/ventral/orthogonal respectively, *p* = *0.54/0.83/0.27*, *n* mice = *8/3/3*, Wilcoxon rank-sum). Despite not reaching group level significance, maximal number of synaptic connections detected in a single recording in the dorsal tangential insertion reached up to *243* as opposed to *65* and *98* in ventral tangential and orthogonal insertion types respectively. In addition, unlike previous reports showing mostly excitatory neurons to inhibitory neurons,^13^^,^^64^ we observed regularly excitatory neurons to excitatory neurons connections. These two types of excitatory connections exhibit different features depending on whether their post-synaptic target is BW or NW neurons. The average peak time for BW-targeted connections was significantly higher (*2.3 ± 1.19 ms, n = 248*) compared to the NW (*1.63 ± 0.66 ms, n = 730*) connections (*p* = *8.16 × 10⁻¹⁸*, Wilcoxon rank-sum). Similarly, average efficacy (eq. to the probability of spike transmission) was higher for NW-targeted connections (*5.72 ± 5.6%*) compared to BW (*3.4 ± 3.39%*) connections (*p* = *4.21 × 10⁻¹⁵*, Wilcoxon rank-sum) while the contribution (eq. to the proportion of post-synaptic spikes locked to pre-synaptic inputs) was modestly lower for NW connections (2.06 ± 2.4%) compared to BW (*2.80 ± 3.8%*) connections (*p* = *0.0041*, Wilcoxon rank sum). Furthermore, we did not observe an overall difference in the distance of the excitatory connections onto NW or BW neurons. Interestingly, we did observe unusual long connection distances beyond 400 μm for both connection types corresponding to inter-region connections (Figures 5D and 5F, *p* = *0.96*, avg. BW/NW = *139 ± 182/129 ± 148 μm*). We found these long-range connections specifically in the recordings encompassing many channels within the pyramidal layer (*1* out of *3* mice) with multiple hippocampal regions showed prominence of local connections, as previously observed.^13^

## Discussion

In this paper, we illustrate a technique of inserting a single NP tangentially along the hippocampal antero-posterior axis to maximize the spatial coverage, in head-fixed mice (Figure 1). Tangential insertions have been done previously in the retinotectal^63^ and thalamo-cortical^65^ visual systems in mice, but the approach has not been tested in the hippocampus.

We hereby highlight the key advantages of this technique including increased channel yield from hippocampus, both from dorsal (whole hippocampus) and ventral (CA1) tangential insertions (Figure 1), allowing for the tracking of LFP patterns with high spatiotemporal precision, as illustrated by ripples (Figure 2); the ability to record a large number of neurons from stratum pyramidal over an extended anatomical range (Figure 3); together with more classical behavioral tracking (Figure 4); the potential of capturing synaptic connections across the extended hippocampal spatial domain (Figure 5). Consequently, this approach opens new avenues in analyzing possible relationships between anatomical patterns, connectivity, LFP, and behavioral variables across the longitudinal axis of hippocampus.

We show via extensive camera tracking correlations between hippocampal neuronal activity and behaviors such as whisking or running.^66^^,^^67^ It is important to highlight that vibrissae movements are rapid, occurring sometimes within *100* *ms*; hence, extracting facial movements below *20 Hz* sampling rate would result in an overestimation of resting state (Figure S7). The animals in the current setup were well habituated, therefore alternating between stable silent and mobile run periods. Running periods, however, were classified based on the output from the treadmill, which was preceded by actual bodily movements in mice, thereby slightly underestimating the actual run bout durations. This is exemplified by earlier onset of theta oscillations compared to the initiation of run bout (Figure 4).

While imaging techniques have also been developed in parallel to record the activity of large numbers of neurons from cortical,^68^^,^^69^^,^^70^ and subcortical regions,^71^ they still lack temporal resolution due to their reliance on voltage- or calcium-sensors and/or the tradeoff between scanning speed and image volume. Furthermore, such studies entail elaborate and more expensive setups, which may not be necessary for most experiments requiring simpler readout. Because NP recordings can also be applied to humans,^72^^,^^73^^,^^74^^,^^75^ knowledge gained from recordings in rodents (as in our case) and other species (e.g.,^76^^,^^77^) can in principle be directly compared to human data, if anatomical differences are considered.

In principle, our approach could also be extended to other probe types and recording conditions. Several approaches have been adapted to maximize the neuronal yield for e.g., insertion of multiple probes in parallel,^78^^,^^79^ multi-shank,^9^ or of high-density probes.^10^^,^^48^^,^^80^ These approaches have been primarily designed to maximize the neural outputs from various brain regions and are not necessarily hippocampal-specific. Usage of multiple probes, however, can be technically challenging due to spatial constraints making physical implementation difficult. Multi-shank probes offer an advantage over single-shank probes, but they still lack the high spatial sampling our approach offers across the longitudinal axis of the hippocampus.

Previously, Swanson et al. used an angular approach with silicon probes. They inserted silicon probes at an angle of *60°* within the coronal plane since their goal was to simultaneously target retrosplenial cortex and dorsal hippocampus. Consequently, their coverage within the pyramidal layer was relatively limited, with just *8–10* channels^81^ compared to our dorsal tangential insertions encompassing more than *100* channels within the pyramidal layer (Figures 2D, 2E, and 2F). Application of orthogonal and ventral tangential insertions seems feasible in freely moving mice, as recently published using a *60°* angle from the posterior midline.^81^ This may be challenging for dorsal tangential insertions in mice as the probe is inserted from the anterior midline at an angle of approximately 30° from the midline plane rendering the implant accessible for the animal, potentially causing mechanical disruption. In addition, this positioning of the NP at a flat angle will make the implant very large and extended toward the frontal part of the head, which may also hinder the head movement in the animals. Previously, feasibility of tangential insertions in the cortex of rats in chronic settings was shown to be possible at an angle of *65°*^82^ suggesting that all tangential hippocampal insertions could be done in rats. Tangential insertions can also be combined with a head-mounted multi-camera system for behavioral tracking in freely moving mice.^83^^,^^84^

In the current study, we have explored only the acute tangential insertions in head-fixed conditions and have shown that they can substantially increase the sampling within the hippocampus compared to more orthogonal insertions, enabling us to capture local field potential dynamics across a larger extent of the longitudinal axis. This facilitated the detection of ripples up to *130* channels corresponding to about 1.3 mm of tissue, allowing the investigation of ripple dynamics including propagation and coordination. Notably, the higher spatial sampling afforded by tangential hippocampal insertions has already revealed non-uniform ripple dynamics within a single event (Figure 2), suggesting that the fine-grained spatiotemporal structure accessible through our approach uncovers propagation phenomena that are less smooth than initially observed via a multi-shank approach.^85^ Previous approaches have looked at ripple variability, finding correlations between hippocampal ripple parameters and cortical activation patterns,^42^^,^^86^^,^^87^^,^^88^^,^^89^^,^^90^ including ripple variability along the hippocampal longitudinal axis approach.^91^ Our method paves the way for further dedicated investigation of detailed ripple dynamics along large stretches of the hippocampus. Combining such an approach in more animals (Table 2), with laminar hippocampal or cortical recordings, optogenetic manipulations, and advanced machine-learning approaches,^92^^,^^93^^,^^94^ will enable us to reveal the functional role of these phenomena (if any), as well as their underlying mechanisms.

In terms of cell and synaptic yield, we did not observe a statistical significance between tangential and orthogonal insertion types. However, maximal cell and synaptic yield were higher for dorsal tangential insertion compared to ventral tangential or orthogonal insertions. It is to be noted that orthogonal insertions were performed at an angle of *45°* from both the sagittal and the coronal plane. Although we did not observe any statistical difference in the cell yield between orthogonal and tangential insertions, our angled approach collectively across all insertion types markedly increased cell yield from hippocampus compared to the purely vertical approach done previously.^8^ Altogether, tangential insertions can improve ripple measures, providing continuous observation of their internal dynamics, as well as cell yields and functional connectivity, which are difficult to optimize concomitantly (Table 2).

Additionally, we observed diverse connectivity profiles including locally connected circuits and more broadly connected networks (Figures 5D and 5E). The lack of statistical significance could be attributed to the limited sampling size and higher variability, possibly stemming from the sensitivity of the placement of the probe right next to the pyramidal layer(s) of the hippocampus. Furthermore, our findings are in line with previous studies regarding the prominence of excitatory monosynaptic connections toward putative inhibitory neurons (Figure 5;^13^^,^^64^). Given the methodological limitations of detecting connectivity and its inconsistency with patch-clamp-based estimates,^95^^,^^96^^,^^97^^,^^98^ it remains to be understood whether these outcomes truly reflect the physiological connectivity.

### Limitations of the study

The lack of statistical significance in certain results of our study should be attributed to the limited sampling size and the particular difficulty to obtain a tangential insertion inserted along the pyramidal layer of the dorsal or ventral hippocampus (Table 2), where higher yield in cell number, connections, and ripple power can be obtained.

## Resource availability

Data will be provided upon request, please refer to Table 2 to pinpoint animal identification.

### Lead contact

Requests for further information and resources should be directed to and will be fulfilled by the lead contact, Jeremie Sibille (jeremie.sibille@college-de-france.fr).

### Materials availability

This study did not generate new unique reagents.

### Data and code availability

• Data: Data reported in this paper will be shared by the lead contact upon request.
• Code: This paper does not report original code.
• Additional information: Any additional information required to reanalyze the data reported in this paper is available from the lead contact upon request.

## Acknowledgments

We would like to acknowledge the entire Schmitz lab for providing support: Roberto de Filippo who wrote the license, Antje Fortströer, Anke Schönherr, and Friedrich Johenning who supported the underlying administrative work. This study was supported by the 10.13039/501100001659German Research Foundation (10.13039/501100001659Deutsche Forschungsgemeinschaft (DFG), projects: 184695641 – SFB 958, 327654276 – SFB 1315, 415914819 – FOR 3004, 431572356, KR 4062/5–1, and under Germany’s Excellence Strategy – Exc-2049-390688087), by the 10.13039/501100002347Federal Ministry of Education and Research (BMBF, SmartAge-project 01GQ1420B) and by the 10.13039/501100000781European Research Council (ERC) under the European Union’s Horizon 2020 research and innovation program (grant agreement No. 810580).

## Author contributions

Conception and design: J. S.; methodology: A. K., J. S.; investigation: A. K., J. S.; writing – original draft: A. K., V. E., J. T., J. S.; writing – review and editing: A. K., J. K., V. E., J. T., D. S., J. S.; funding acquisition: J. K., D. S.; resources: J. K., D. S.; supervision: J. K., D. S., J. S.

## Declaration of interests

The authors declare no competing interest.

## Declaration of generative AI and AI-assisted technologies in the manuscript preparation process

No AI has been used in the writing process of this article.

## STAR★Methods

### Key resources table

**Table:** 

REAGENT or RESOURCE	SOURCE	IDENTIFIER
**Chemicals,peptides, andrecombinantproteins**		

Isoflurane NaCl 0.9% Lidocaine Metamizol Ketamine Xylavet Novaminsulfon DiI	Cp pharma Braun (Germany) Braun (Germany) Sigma Aldrich (Germany) Ketabel Cp pharma Ratiopharm Abcam-ab	5896148959197 2737756 03676850 06882768 19487223 1205 PZN-06882768 145311

**Experimental models:Organisms/strains**		

C57BL6/J (Male, 2–4 Months)	Charité	IMSR_JAX:000664

**Software andalgorithms**		

MATLAB	MATLAB ©	N/A
Open-Ephys	https://open-ephys.org/	N/A
Python	https://www.anaconda.com/	N/A

### Experimental model and study participant details

#### Experimental model

All data presented were recorded in head-fixed conditions, with a protocol that was approved beforehand by the corresponding authority (Landesamt für Gesundheit und Soziales Berlin – G0298/18). Maximum care is dedicated to minimizing the number of animals used (*n* = 14 Adult C57BL/6J male mice from different litters) which all stem from local breeding facilities (Charité-Forschungseinrichtung für Experimentelle Medizin, started from the IMSR_JAX:000664 line). The use of only male animals limits the generalizability of this study.

#### Animal surgery

A head post was implanted ∼3 weeks prior to the recording day. Such implantations were performed in a Narishige stereotactic frame using isoflurane (2.5% in oxygen; Cp-Pharma G227L19A). Analgesia was delivered to the animal 1 day prior and 3 days post-surgical operations (Metamizol diluted in drinking water, 200 mg/kg body weight; Zentiva-Novaminsulfon). It is important to note that when the head-post was fixed, it was combined with a large dental cement-based crown (Paladur, Kulzer) to better fix the head-post on the skull, permitting a robust preparation that will stay over the 3–4 weeks of consequent manipulations. This crown was set up to grasp the lateral ridges of the skull and the frontal bone, to maximize stability. To facilitate dorsal tangential insertions which are relatively flat, it is important to reduce the crown height to 1–2 mm on the opposite side from the planned insertion. This crown was also made to surround the skull to hold fresh PBS during recording for grounding purposes. Mice were gradually habituated to head fixations on a running band (Phenosys©, Speedbelt 1601–0102, analog output 0–5 mV) for over three weeks, including breaks of a few days and monitoring of their wellbeing.

A second surgery was performed on the recording day to drill the craniotomy and locally remove the dura. A recovery period of at least 2 h was given before recordings, and the wellness of the animals was assessed before initiating the recordings. The recordings were performed the same day as craniotomy to minimize the exposure of the tissue. Craniotomy, however, can also be performed a day prior to recording which improves the animal’s recovery from anesthesia. The craniotomy was performed with the following coordinates: −3.5 to −2.5 mm AP, 0 to +1 mm ML from bregma for dorsal tangential insertions and −4 to −3 mm AP, +2 to +3 mm ML from bregma for ventral tangential insertions. The craniotomy for tangential dorsal insertion was purposely made larger to allow easier (re)insertion. During the craniotomy surgery, we used the drill to carve into the dental-cement crown and add a grounding wire, fixing it to the crown with extra dental cement, such that one end touched the skull within the crown while the other extended 3–5 cm in the air. The exposed grounding wire end was later used to connect to the NP external reference directly, which can be equipped with corresponding gold pins. For efficient noise reduction, the ground wire was immersed in the crown containing PBS. This ground wire replaced the classical screw in the skull above the cerebellum classically done for chronic implant. During the recordings, we prevented the bath from drying out by regularly refilling it with fresh PBS.

### Method details

#### Insertion and staining

It is advised to prepare the micromanipulator’s placement beforehand (inserting with dummy probe for placement testing) to minimize the head-fixation time in the set-up during recording day. The micromanipulator arm (New Scale) was positioned on a custom Thorlabs mounting platform to realize an overhead approach, which can also be done using a New Scale ring. The New Scale manipulator was positioned at an angle of 30° from the sagittal plane for both dorsal and ventral insertions. Using the rotating capabilities of New Scale arm, the moving head could be placed with the aligned probe either with a 30° angle from the interaural plane for dorsal insertions, or 60° for ventral insertions (Figures 1A, 1B, and S1C). It is to be noted that our orthogonal insertions are performed at an angle of 45° from both the sagittal and the coronal plane (cf. Figure 1), leading to higher unit yields compared to more standard purely vertical insertions (Table 2;^8^^,^^79^). Given the anatomy of hippocampus, angular insertion along the longitudinal axis encompasses relatively more cortical tissue compared to vertical ones, hence any deviation at the insertion point may amplify the errors in targeting the precise sub-structure of the hippocampus. Pre-recording practices, great attention to micromanipulator’s arm placement, attentive care on the skull flatness during head-post implant, and pilot brain slicing to confirm the probe placement will be considered as must-do prior to recording day. In addition, it is important to probe the possible variability between different mouse lines or ages to succeed with reproducible hippocampal tangential insertions. It is to be noticed that for tangential insertions, probes were inserted 5 to 6 mm deep in the tissue (particularly for ventral and dorsal insertion) to reach the full extent of the hippocampal formation, recording only from the lower shank of the NP. Once the probe is placed, the neuronal synchrony and LFP profile should be thoroughly checked to infer whether slight probes repositioning in different entry points of the craniotomy (Figure S1C) would putatively improve the desired alignment. Acquiring repeated recordings on the same set-up will improve reproducibility. In our hands, upon initial attempts, up to three re-insertions were required to reach a satisfying LFP profile, exhibiting ripples across a large extent of the probe. On consecutive days, satisfying positioning of the probe within the hippocampal formation was obtained on the first attempt, thanks to strict monitoring of the arm angle, careful placement of the animal, and cautious assessment of the head flatness relative to the head-post implant. Despite these precautions, our yields varied considerably from one recording to the next (Table 2), suggesting that the concomitant detection of ripples, cells, and functional connectivity is only possible within a narrow window. Tangential insertions have been used in the visual pathway, specifically in the superior colliculus^99^^,^^100^ and visual cortex^65^^,^^101^ to increase the quantity of measured tissue. Building on this non-conventional approach, we applied it to the hippocampus with a focus on hippocampal ripples.

On recording day, the tangential hippocampal insertions were initiated by slowly approaching the craniotomy and then moving carefully in the brain for at least 4–6 mm, followed by a withdrawal of 50–100 μm to release accumulated mechanical pressure. For the dorsal tangential insertions, given its flat angle, it is very important to avoid having any PBS in the crown in the beginning of the insertion in the tissue, otherwise the surface tension of the water will add extra bending of the probe, with an additional 10–15°, reducing reproducibility. It is therefore strongly advised to temporarily remove the grounding solution (PBS) at the time of contact of the probe with the brain tissue to guarantee an insertion aligned with the arm’s angles. Lastly, measuring bregma and Lambda positions with the NP probe tip once the New Scale arm is in position can give extra measures for later positioning confirmation and ultimately better reproducibility.

As the probe is moved within the tissue, the presence of ripples and/or a large amount of spiking activity should be used to monitor the presence of CA1 or CA3 pyramidal layers along the channels (Figures 1C and 2). This information may play an important role in assessing whether reinsertion at a different location, angle, or depth of the probe will be needed (Sup. Figure 1). Importantly, given the proposed angle, the distance from the brain surface to CA1 can exceed 1.5 mm; therefore, a minimum of 5 mm of insertion should be done for ventral and dorsal tangential insertions (Figures 1 and S1). Once a satisfying location was obtained, the probe was left to settle in the tissue for ∼30 min before starting the recording. The NP probe was coated with DiI (Abcam-ab 145311, in EtOH) before insertion. Once the recording was finished, the mice were sacrificed with a lethal injection of ketamine-xylazine (100 and 15 mg per kg body weight, respectively), before proceeding with cardiac perfusion via PBS and paraformaldehyde (PFA). Brains were left in PFA overnight, sliced coronally (100 μm) and mounted (DAPI-Fluoromount-G Biozol Cat. 0100–20). Mounted slices were pictured, and the probe’s track was 3D reconstructed using SHARP-Track^102^ to relocate each single channel within the Allen Mouse Brain Common Coordinate Framework (Figure 1).^103^ Once the reconstruction was finished, only the channels, the cells, and the synaptic connections within the hippocampal formation (CA1-2-3 summarized as “CAX”, DG-mo-sg-po summarized as “DG”, ProS, alv, dhc, HPF, SUB, cf. Figure 1; Table 1) were kept for further analysis. The number of channels positioned within the hippocampal formation should serve as a benchmark for successfully reproducible tangential insertion. Similar positioning of hundreds of channels in the hippocampus can also be optimized using orthogonal multi-shank insertion e.g.,^48^ albeit at a roughly 10-fold lower spatial resolution along the longitudinal axis (i.e., the spacing between the shanks, rather than the spacing between recording sites), and at a significantly higher cost (single-shank probes compared to multi-shank). Open-Ephys acquisition software (www.open-ephys.org) in combination with a PXIe system (National Instrument NI-PXIe-1071) was used to record NP probe signals (NP 1.0, IMEC).

#### Data acquisition and local field potential extraction and coherence

LFP traces were recorded from the 384 channels of the Neuropixels probes (NP 1.0, sampled at 2.5 kHz). To analyze the prominence of different frequencies, power spectral density (PSD) was computed using Welch’s method with a window size of 4 s and 50% overlap. Power of theta, gamma, and ripple was obtained by summing the PSD within different frequency ranges (Theta: 4–10 Hz, Gamma: 30–100 Hz, Ripple: 110–240 Hz, Figures 1C and 2A).

For a few analyzed dorsal tangential insertions (*n* = 6), theta and gamma power revealed large areas of the probes showing theta/gamma modulation during running movements (Figures 4D and S4, cf. Rev;^104^). To assess the spatial distribution of activity along the probes, we computed the current source density (CSD) for each recording by taking the second spatial derivative of averaged non-overlapping 1000 ms bouts of activity time-locked to theta-trough (Sup. Figure 2). Because of the high spatial resolution we optimized within regions, a large channel separation (10 channels) was used in order to reduce the effects of volume conduction. To further probe the spatial distribution of theta activity, we computed PSD using FFT in a representative 20 s long running epoch across all the channels (Figure S3). Summing the power in theta band (5–10 Hz) revealed a non-uniform pattern of theta activity across the probe (Figure S3A, middle panel). Consistently, PSD traces from chosen channels (1-deep, 150-intermediate, and 300-superficial) (Figure S3A, lower panel) show a similar gradient of theta activity. To visualize the full theta spectrum, we also constructed a frequency-by-channel power map to confirm the localization of theta prominence in the intermediate channels (Figure S3B).

Using power spectrum extraction and its related normalized power, it became possible to compare the different profiles along the 384 channels from MUA, theta/gamma modulation, cell count, and anatomy (Figure 1C). This alignment revealed that the probe can lie within the CA1 area and capture either the pyramidal layers, or the theta/gamma oscillations, or both, depending on its position below the pyramidal layers. As illustrated by other laboratories, theta/gamma oscillations are more prominent in the radiatum and lacunosum, compared to pyramidal layers where the ripple power is at its maximum.^48^ Coherent activity in the theta/gamma band was also shown to extend over similar areas of the probe, as was theta-gamma coupling during running movement (Figures S3 and S4). Apart from arbitrary frequency bands, spectrogram analyses were performed using the Stockwell transform,^105^ which utilizes a sliding window to allow comparable resolution across frequencies (0–250 Hz in this case) for time-varying signals (Figures 2C and 4C).

#### Ripple detection and propagation analysis

Before pursuing finer analysis on ripple propagation, it is important to note that NP 1.0 is equipped with 32 analog-digital converters (ADCs) multiplexing 12 channels each, which are sequentially sampled. This results in an inter-channel sampling offset of 33.33 μs which cumulates across channels within an ADC leading to an overall lag of 399.96 μs (∼0.4 ms) between first and the last channel of an ADC. It is important to correct these lags prior to ripple detection.

For ripple propagation detection, LFP was bandpass filtered in the range of 110–240 Hz using 4^th^ order Butterworth filter. The filtered trace was then Hilbert transformed to capture the ripple power which is then shown by its absolute value. Putative events were classified as ripples if the magnitude of the Hilbert transform exceeded a threshold of 5× SD. Events shorter than 50 ms or longer than 200 ms were discarded. The ripple examples shown are from a dorsal tangential insertion (Figures 2C–2H). Ripple activity was prominent across more than a hundred channels showing the extended localization of ripple events. The given figure, however, shows every 4^th^ channel to avoid delays originating from the NP1.0 checkerboard pattern of the channels on the probe (Figures 2E–2H). To assess the ripple propagation using standard protocol, Hilbert amplitude envelope of ripple band filtered signal was computed and normalized to the pre-ripple window. The time point crossing 50% of the maximum normalized Hilbert envelope was computed across each channel which showed a trend of bidirectional ripple propagation. To further extract the direction and speed of the propagation, a linear polynomial was fitted over the peak ripple power latencies across all the subsequent channels. We further complemented the Hilbert envelope analysis with peak-by-peak analysis. The time points corresponding to each ripple cycle were assessed across channels thereby providing a finer readout of oscillatory activity across the extended tissue. Peak-by-peak analysis revealed a more heterogeneous propagation pattern at the level of single oscillation with no dominant directionality (Figure 2).

#### Spike sorting, quality metrics

Spike sorting was done using Kilosort 2.5 (Figure 4;^106^). Standard cluster quality metrics were calculated to remove units of bad quality (Figure 3E, Isolation distance >10, refractory period violation (ISI) < 0.05%). All single-units were manually cured to search for over-clustered neuronal waveforms on Phy2.^107^ Once curing and quality metrics were applied, waveforms were locally recalculated by averaging the raw NP data at all spike times from this single-unit (up to 50 000 spikes, when possible, Figure 5D). These recalculated waveforms should also include compensations for the slight offset correction of 2.78 μs between neighboring channels within the same analog-digital-converter, as previously done.^63^^,^^65^^,^^100^ This offset correction should be distinguished from the offset correction done in the LFP extraction for the ripple analysis (cf. above). In addition, to finalize the manual curing, a custom-written Graphical User Interface (GUI) was made to label the different single-units as good based on: 1 proper waveforms shape; 2 the absence of a prominent peak in the ±0.1 ms lag in its ACG; and 3 the absence of a peak in the ±0.3 ms lag in the CCG with all possible other combinations with other single-units.

When tangential insertions are properly placed adjacent to the pyramidal layer, cell yields can reach the hundreds (4/14 recordings, Table 2), with up to 2 neurons per channel. Combined with ripple and synaptic yields, this provides a promising framework for future studies. Notably, similar yields can be optimized independently, in ripple monitoring,^108^ or using more expensive multi-shank insertions.^48^ Finally, an approximate distinction between putative excitatory versus putative inhibitory neurons was based on the shape of their waveforms at the channels of biggest amplitude (usually named best channel). To do so, we split waveforms at 0.42 ms of their peak-to-trough duration to distinguish between broad waveforms neurons (BW) and narrow waveforms neurons (NW), as a crude approximation of putative excitatory and inhibitory neurons respectively (cf. Figure S6). According to a recent report using channel rhodopsin based optotagging,^109^ it is likely that our broad waveform neurons may also include Vip and Id2 inhibitory neurons which have larger waveforms. The use of artificial intelligence,^110^ combined with large-scale datasets now available online,^10^^,^^56^^,^^111^ may provide solutions for such approximate classifications.

#### Running and facial movements sampled at high frequency

Head-fixed mice were placed on a running band (Figure 4A). For time alignment purposes, the different time triggered logic (TTL) pulses were all recorded on an extra NI-DAQ card (PXI-8381) connected to the NI-PXI recording system. These TTL included the 1 s long regular TTL pulse from the NP recording card, the camera frame triggers (at 150 to 200 Hz), and the analog signal from the running band (continuously recorded at 30 to 40 kHz). For all recordings, 1 camera (Basler acA1920-155c) was placed to monitor facial movements (Figures 4B and S7, which can be improved to 3 to 5 cameras to monitor different sides of the face, and pupil of the animal). Illumination was done with IR lamps (ACL25416, Thorlab), equipped with a condenser Lens (Aspheric ACL25416U-B, Thorlab), positioned on the recorded side of the animal body, focused on the face. A separate computer was used to record the camera synchronously, which was externally triggered for each frame via a TTL pulse generated at 150–200 Hz by a dedicated locally made python script. Once recordings were finished, the movement data were extracted from the video using the singular value decomposition (SVD) from Facemap^112^ to plot vibrissae movement for each frame, allowing us to isolate moments of higher whisking activity versus inactive periods (among other facial movement, Figure S7C). In addition, despite mice being head-fixed, regular run bouts could be extracted from the analog run signal, which occurred less often (Fig. Sup. 7C: 2324 run events in 8 mice and 5817 whisking events in 14 mice), likely due to the lower sensitivity of band to the animal’s movements, which may reduce the detection efficiency of running events compared to un-constrained whisking events. Furthermore, whisking events were shorter in duration (Fig. Sup. 7C, averages FM = 300 ± 527 ms, n mice = 14, averages run = 967 ± 2067 ms, n mice = 8, *p* = 1.98∗10ˆ-159, Wilcoxon signed rank test). Putative run/whisker bouts were extracted based on a threshold of 3 X SD of the movement trace. To account for artefact-related detections, bouts shorter than 10 ms were removed for further analysis. There was an expected increase in theta power during the movement bouts (Figure 4C). Next, we analyzed the hippocampal neuronal correlates of whisking (Figure 4E) and running behavior (Figure 4F). Spiking activity at the level of single-units was assessed 500 ms before and after the onset and offset of movement trials. We found an overall increase and decrease in the spiking activity after the movement onset and offset respectively for both whisking and running. To quantify the proportions of neurons contributing to the change in the firing, a threshold of ±3 X SD of spiking activity after the movement onset/offset was used.

#### Functional synaptic connectivity detection and quantification

Connectivity in the hippocampus between different neurons was addressed using cross-correlation analysis (CCG), using pycorrelat^113^ similarly to what was done in the visual system^99^^,^^100^ and in the hippocampus.^13^^,^^64^

Neurons having less than 2000 spikes were discarded from CCG analysis. CCG was calculated with a bin size of 0.1 ms. A copy of the spike trains, referred to as spike jitters was estimated by randomizing every spike within a 10 ms window, which was then subtracted from the raw CCG. This step, referred to as CCG-jitter correction, removes the putative common input biases which may not be related to mono-synaptic delays. Excitatory contacts were identified by a prominent peak in monosynaptic delays (0.5–3 ms), as done previously.^96^ Putative inhibitory connections were not considered due to the difficulty of detection in the corresponding CCGs. For every pair, different properties were extracted such as the relative distance between the pre-synaptic and post-synaptic neurons, computed by Euclidean distance between their respective best channels (Figure 5F). Because we observed cases of multiple contacts emerging from a single neuron toward several other neurons, or inversely multiple neurons converging toward a neuron, we calculated divergence and convergence of synaptic contacts (Figure 5F). To estimate the synaptic efficacy of each pair (Figure 5F), the baseline of the CCG (−5 ms–0 ms) was subtracted from the area of their CCG peak (0.5 ms–3 ms). To estimate synaptic efficacy and contribution, it is important to avoid using jitter-corrected CCGs, because they can contain negative values which will lead to an underestimation of efficacy values. Synaptic efficacy was defined as the ratio of the peak area (baseline corrected) and the pre-synaptic spike number, while contribution was defined as the ratio of the peak areas (baseline corrected) to the post-synaptic number for the post-to-pre recalculated CCG.

### Quantification and statistical analysis

All quantification and analysis were either done using Python 3 (www.anaconda.com, Figures 3 and 4), or MATLAB (www.matlab.com, Figures 1 and 2), using Wilcoxon tests. “∗∗∗” corresponds to a *p*-value below 0.001, “∗∗”a *p*-value below 0.01, and “∗” a *p*-value below 0.05. For most analysis all 14 male animals were sampled depending on the insertion direction unless otherwise stated.
