# Supplementary material for: Hippocampal tangential insertions of high-density silicon probes in head- fixed mice enhance spatial sampling
Source: iScience. 2026 Nov 5;29(11):118131. doi: 10.1016/j.isci.2026.118131 (PMC13769017; doi:10.1016/j.isci.2026.118131)
Supplement: Supplementary file 1 [file mmc1.pdf]

iScience, Volume *29*

Supplemental information

Hippocampal tangential insertions of high-density silicon probes in head-fixed mice enhance spatial sampling

Annu Kala, Vera Evander, John J. Tukker, Jens Kremkow, Dietmar Schmitz, and Jérémie Sibille

Supplementary figures

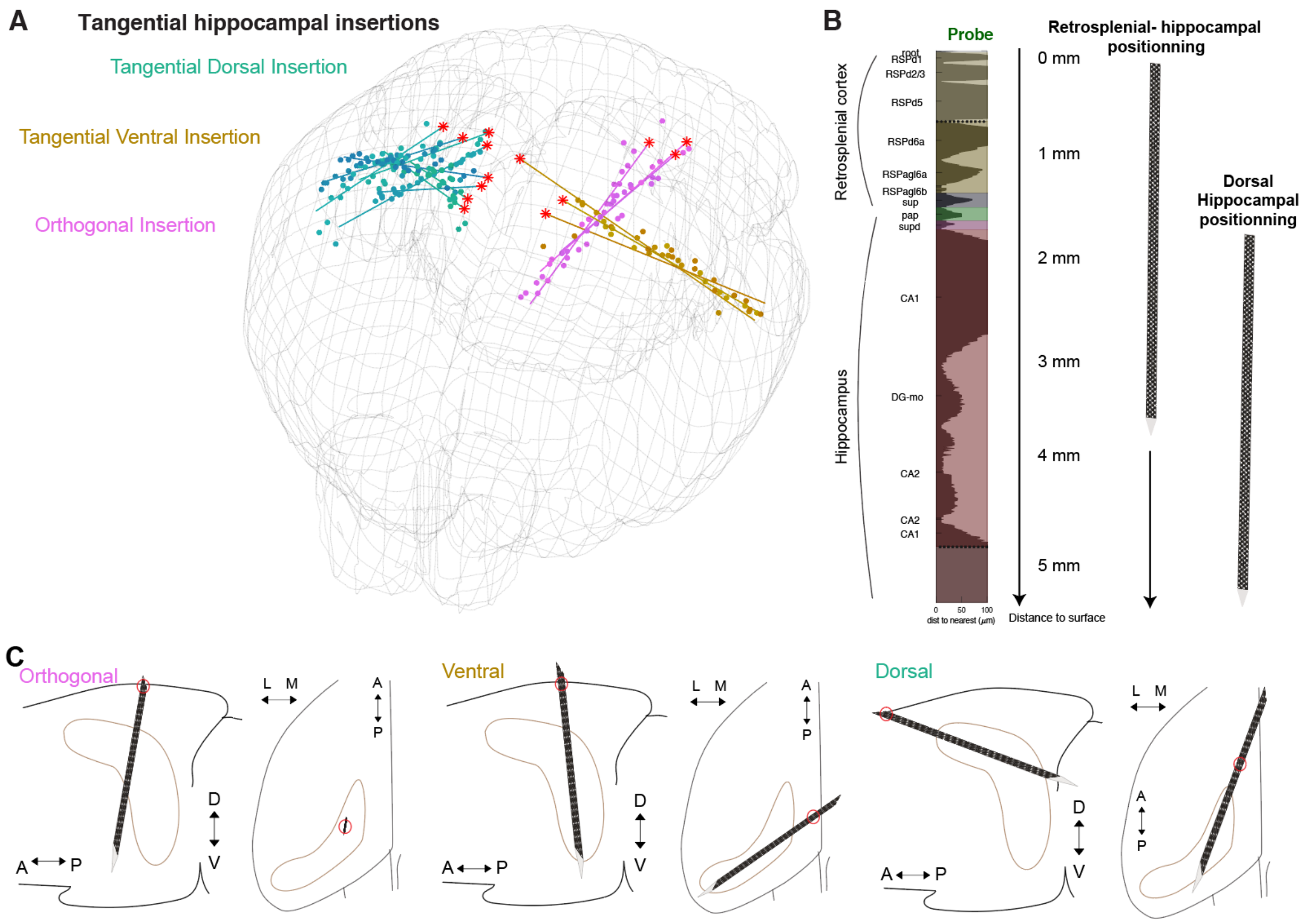

Supplementary Figure 1: Dorsal and ventral tangential insertions require deep insertions to reach the hippocampus: **A.** Schematic of tangential (dorsal and ventral) and orthogonal insertions (n = 14 mice). **B.** The 5 mm track of the dorsal tangential insertion spans several bran regions (left), where two different positions can be realized: a Retrosplenial-hippocampal positioning (middle), or a full hippocampal positioning (right). **C.** Sagittal and interaural planes represent the position of the insertion for orthogonal (left), ventral (middle), and dorsal (right) insertions. Note that comparatively the point of entering the tissue (red circle) is quite variable from one insertion to the other (interaural view).

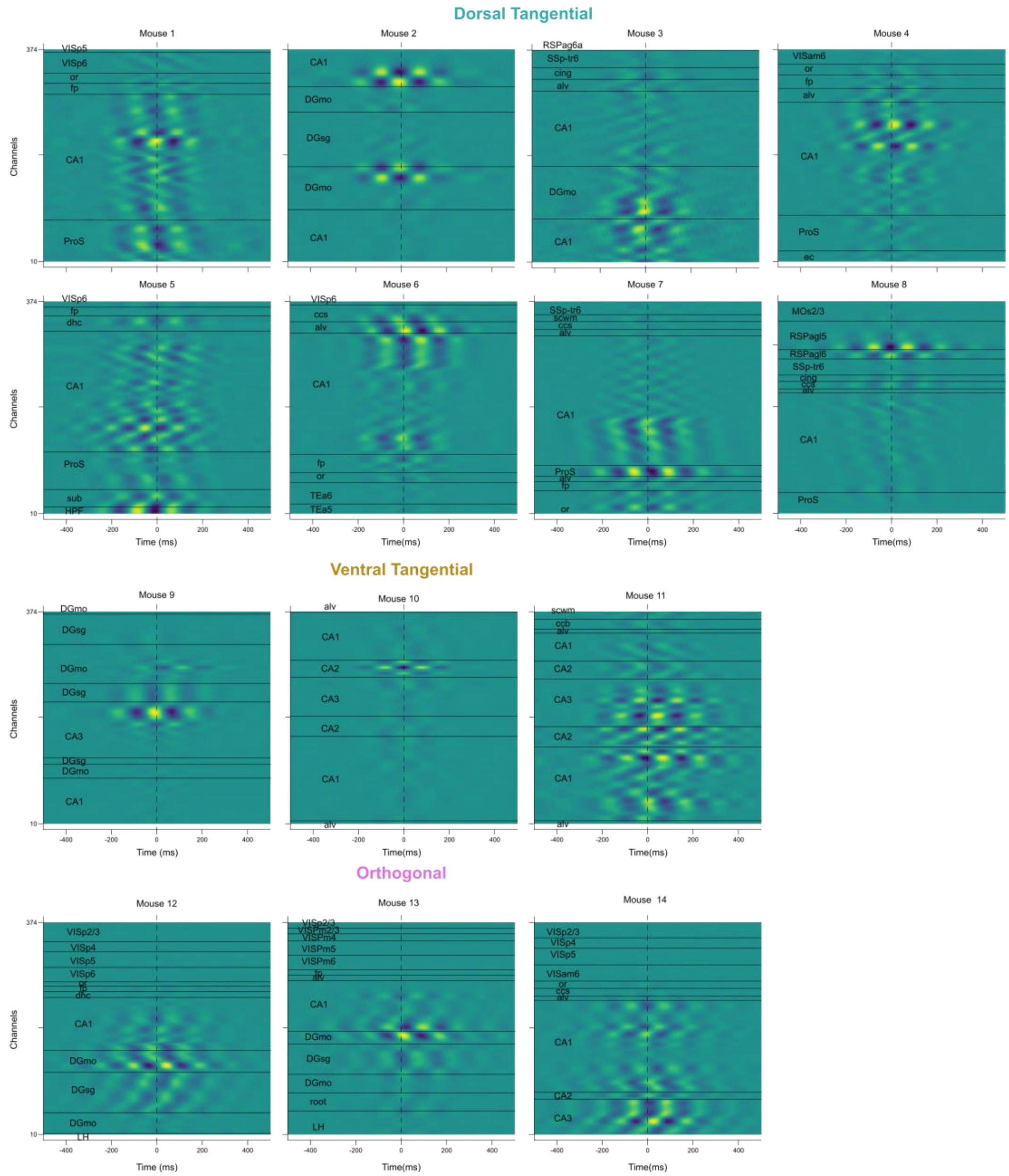

Supplementary Figure 2: Single recording CSD aligned to theta trough. CSD calculated along each probe aligned to theta trough. Timestamps for theta trough were taken from the channel with the highest theta power for each recording. List of anatomical abbreviations can be found in supplementary table 2.

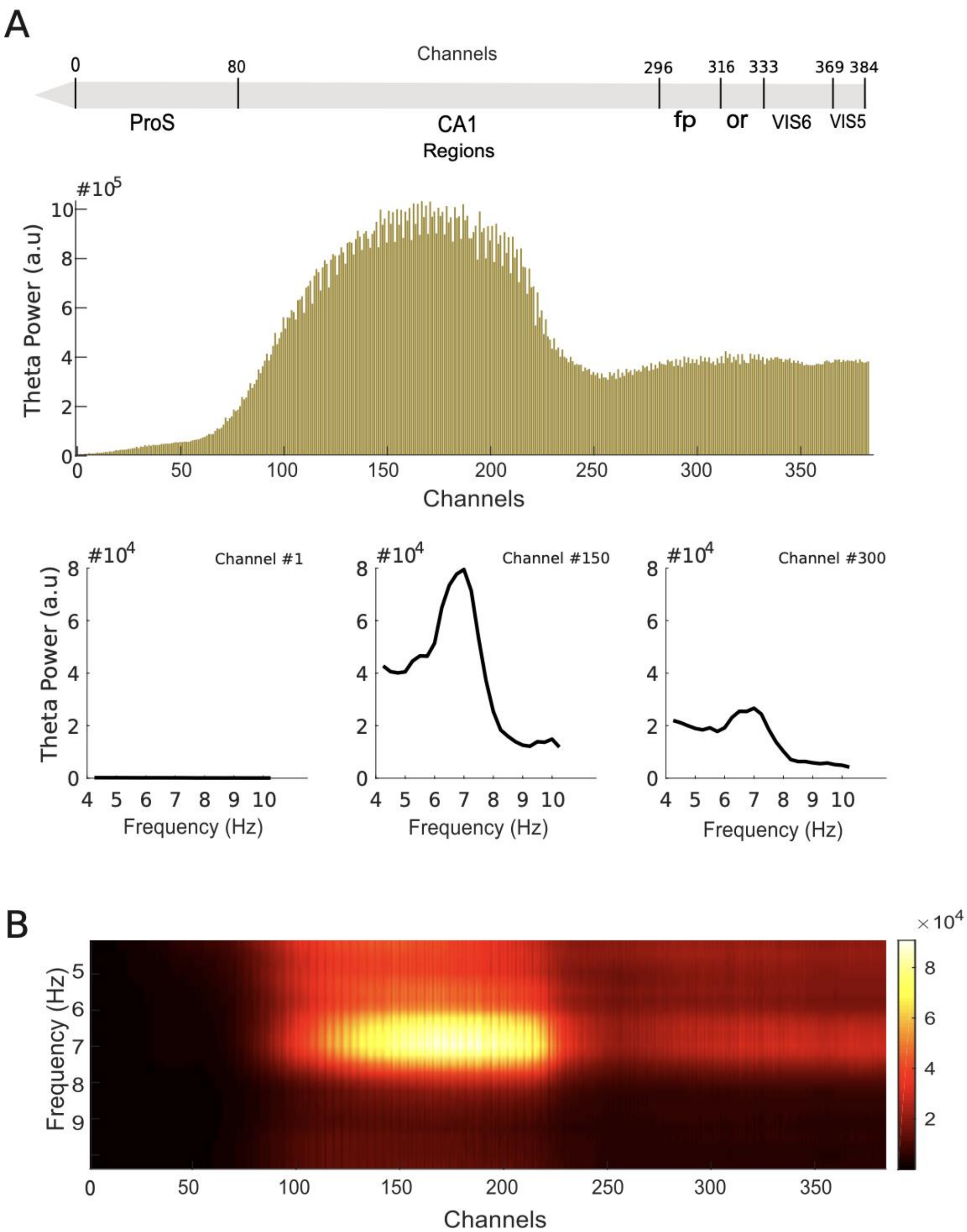

Supplementary Figure 3: Differential theta power distribution across NP during a 20 s long running movement epoch **A.** Anatomical coverage across channels in a representative recording (Top). ProS - Presubiculum, CA1 - Cornu Ammonis 1 (hippocampal CA1 pyramidal layer), fp – corpus callosum posterior forceps, or - Stratum oriens, VIS6 - Primary visual cortex, layer 6, VIS5 - Primary visual cortex, layer 5. Histogram of summed theta-band power (4–10 Hz) computed using FFT across all recording channels during the 20 s running epoch (Middle). PSD traces from three representative channels in deep (channel 1), middle (channel 150) and superficial (channel 300) locations of the probe (Bottom). **B.** Power frequency representation across the entire probe. The heatmap shows power as a function of frequency (y-axis) and channel depth (x-axis), demonstrating spatially localized theta-band activity.

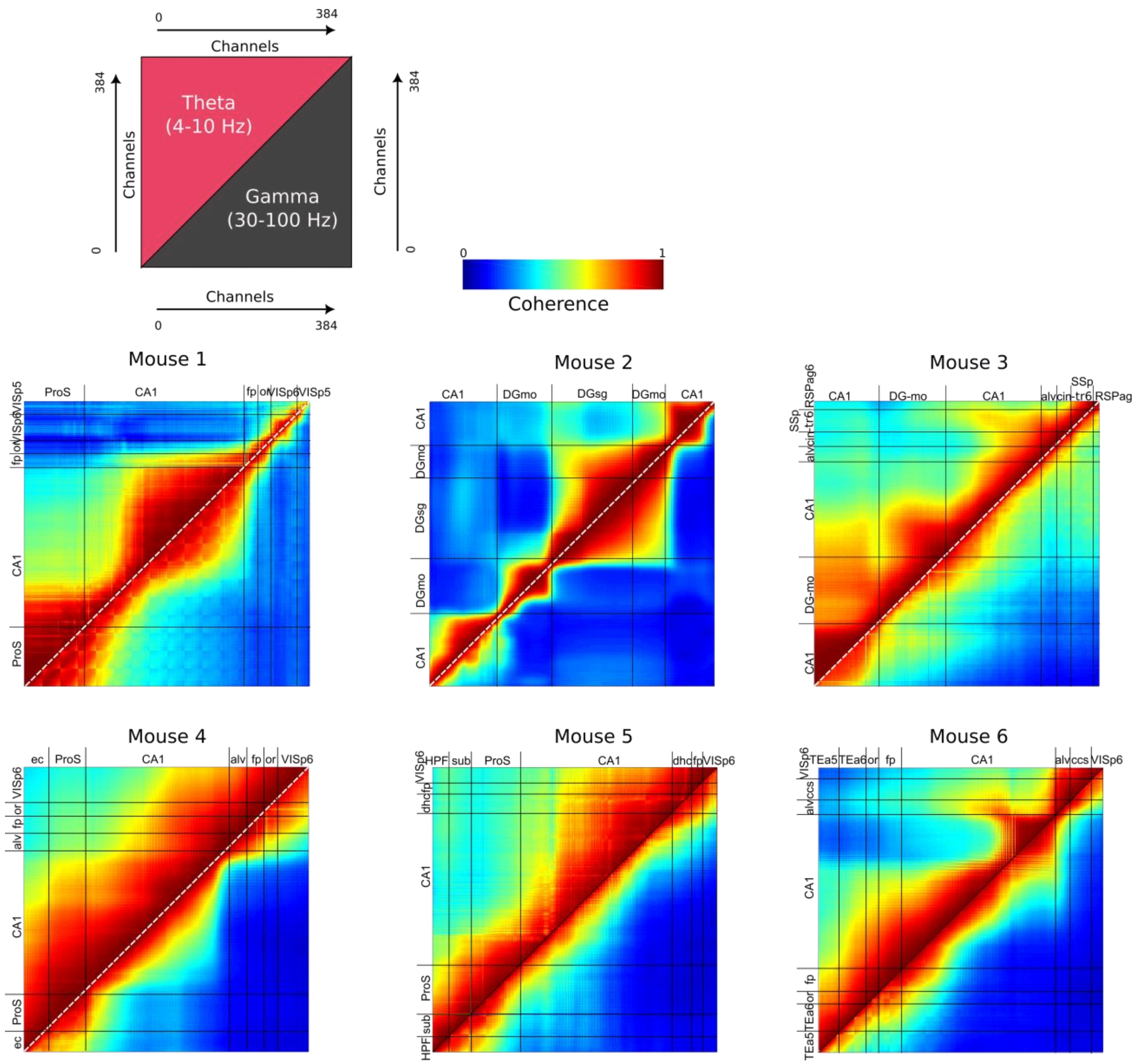

Supplementary Figure 4: Movement-induced theta and gamma power increase Scheme of the coherence quantification arranged along the probe channels (Top). All heatmaps show coherence along the 384 recording channels, from six different dorsal tangential recordings. Colors indicate the magnitude of squared coherence between two channels during movement, splitting theta (top-triangle) from gamma (bottom triangle) range. Brain regions are indicated in the top and left sides for each recording.

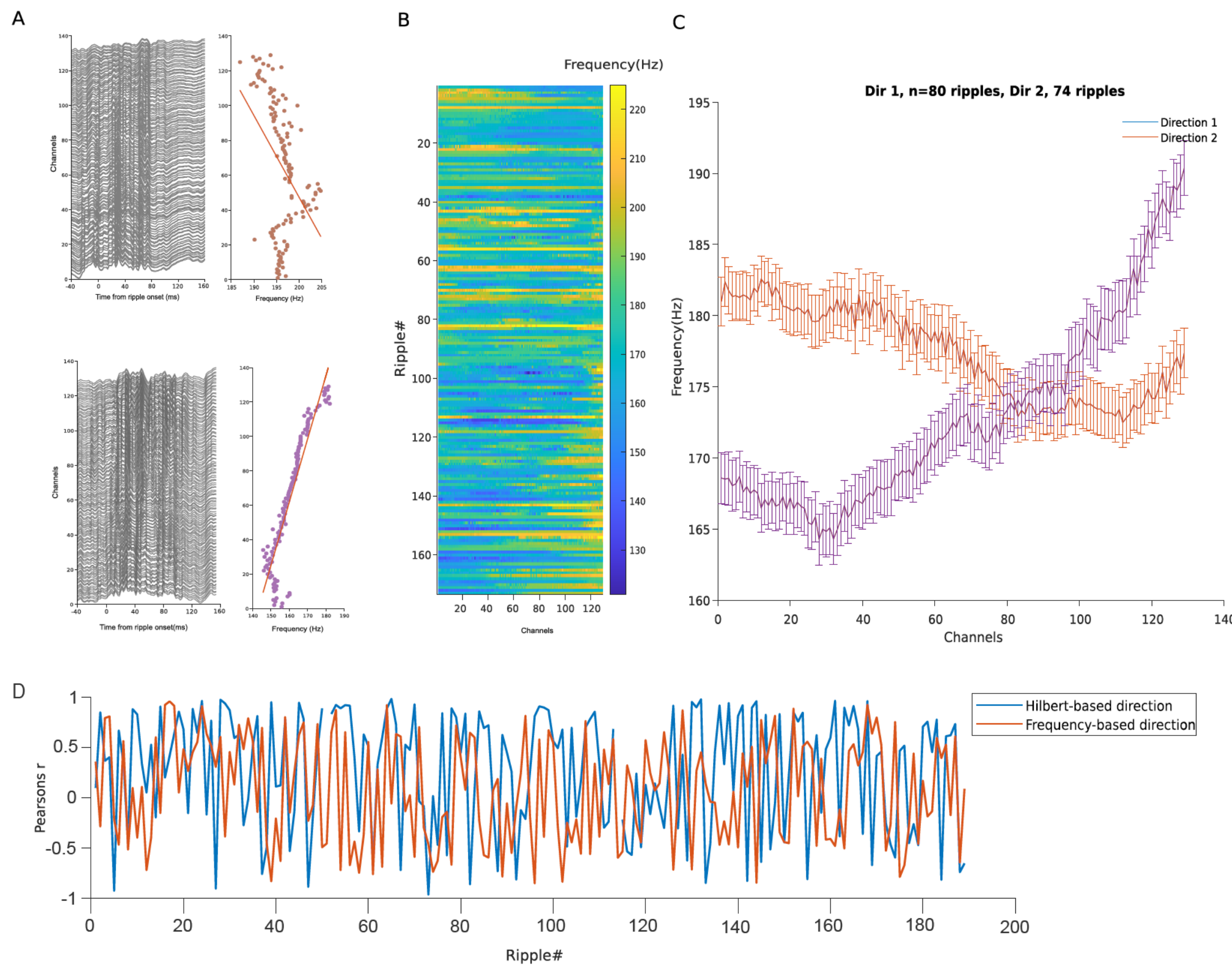

Supplemental Figure 5: Spatiotemporal organization of ripple frequencies across the hippocampal longitudinal axis. A. Shows two representative ripple examples (Upper and lower panels). The left panel shows raw peri-ripple LFP traces. Scatter plots in right panel show channel resolved ripple frequencies using Morlet wavelet. The red lines show linear regression fits of frequencies across channels. **B**. Represents channel resolved heat maps of the frequencies of all putative ripples sorted based on Pearson's directionality index. Note that many ripples displayed a gradient of their frequencies across channels. **C**. Average ripple frequency profiles of opposite frequency gradients. Error bars indicate standard errors of the mean across ripple frequencies. **D.** Pearson's r values derived from ripple band envelope-based power and ripple frequency-based directionalities, shown for all detected ripples from n=3 mice. Note a lack of correlation between these two values.

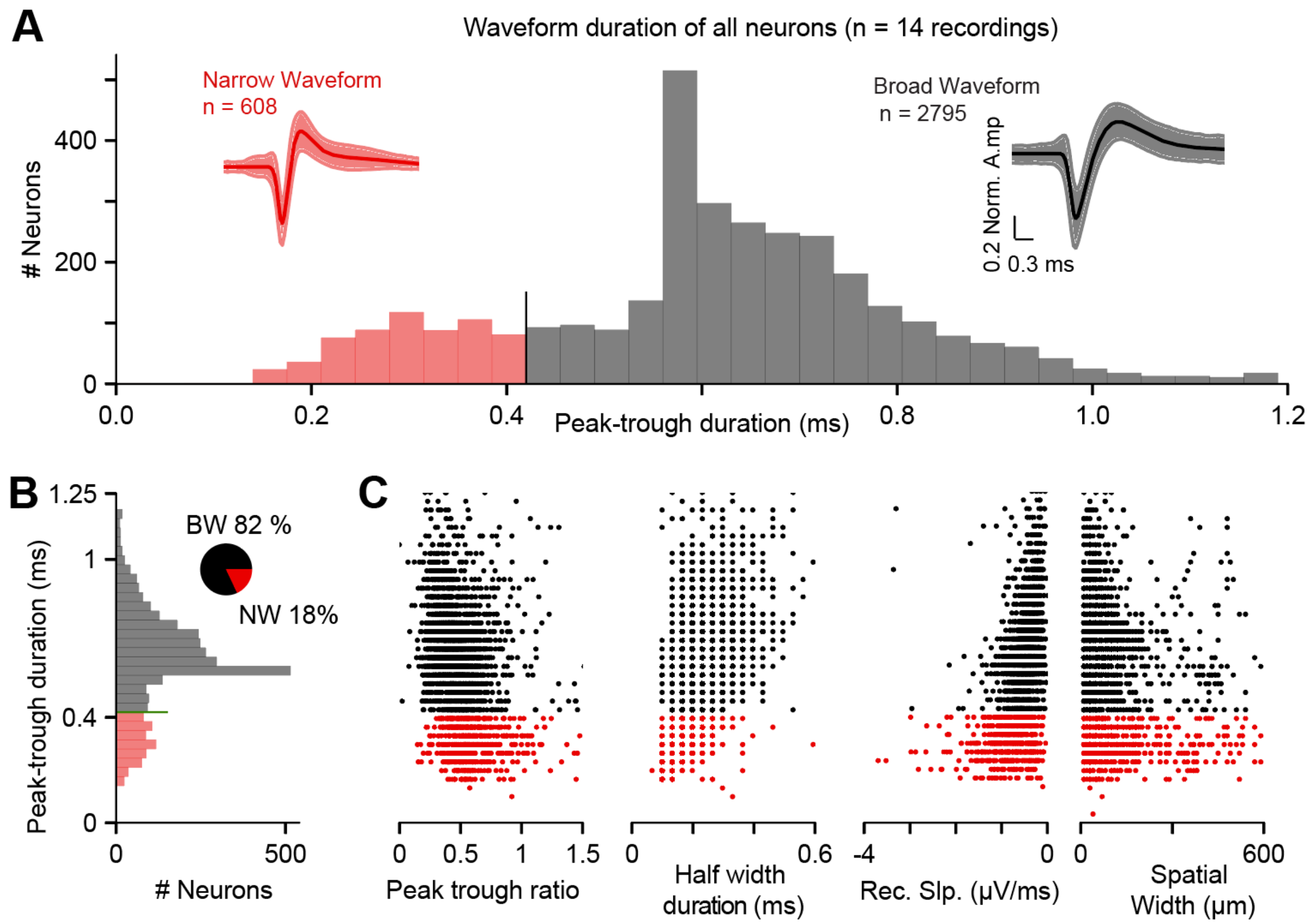

<u>Supplementary Figure 6: Narrow versus broad waveforms neurons in the hippocampus: an approximation to extract excitatory from inhibitory neurons.</u> **A.** Typical peak-to-trough duration used to separate narrow waveforms (NW, red) from broad waveform (BW, black) neurons. Insets show the averages and std of the normalized waveforms from all neurons; scale bar applies to both inset identically. **B.** Peak-to-trough duration plotted vertically, aligned to other waveforms properties. **C.** Quantifications in NW versus BW neurons in the entire dataset; each dot represents either a BW (black) or NW (red) neuron. From left to right: peak-trough ratio, half width duration, recovery slope, and waveforms' spatial width; all sharing the same y-axis as in B. Note that the 30 kHz sampling rate of NP give these incontiguous lines rendering in the different panels.

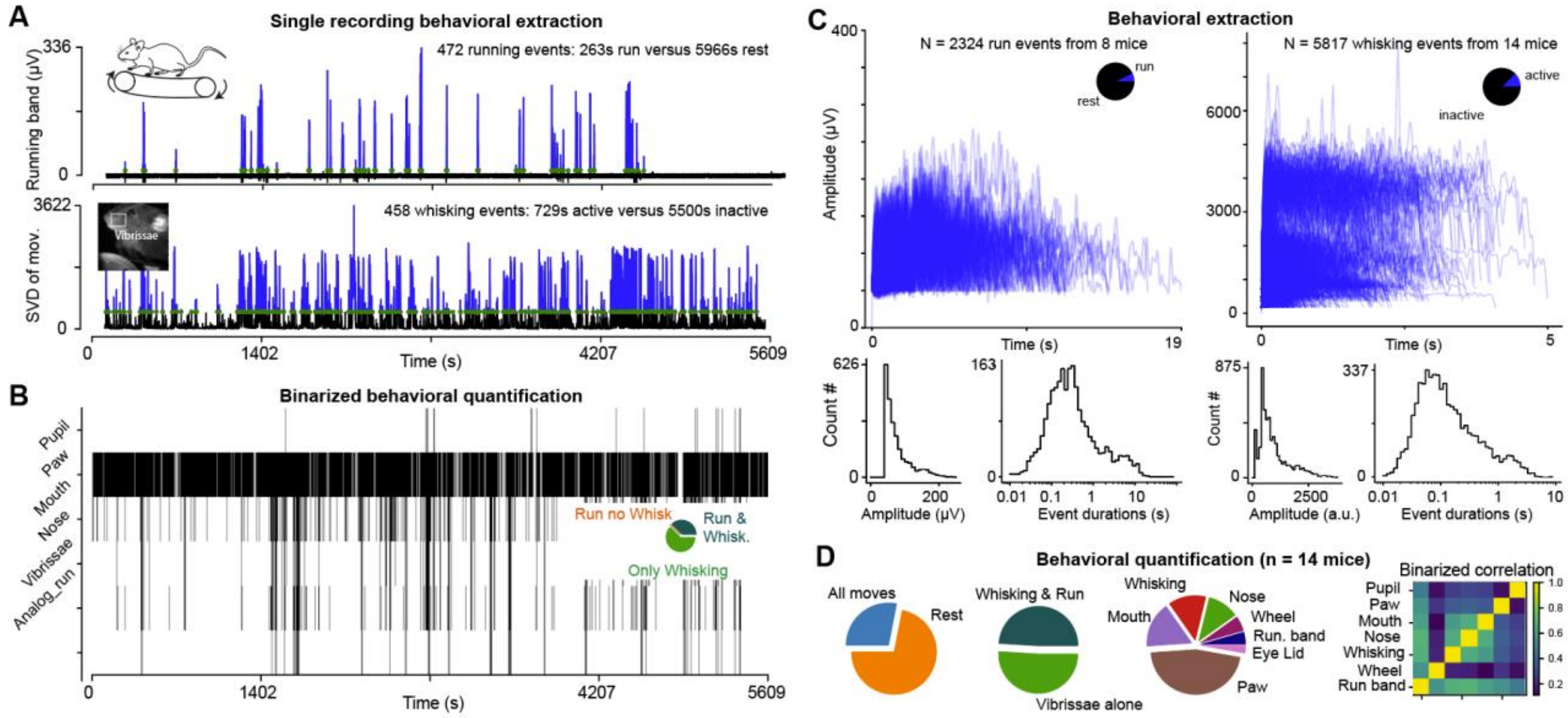

Supplementary Figure 7: Spontaneous behavior (run and whisking) monitored at high frequency. A. Representative recording showing outputs from the running band (top) and singular value decomposition (SVD) signal from the facial movements (bottom, extracted with Facemap). Blue lines indicate the signal above the threshold selected for further analysis; black lines are sub-threshold; green dots indicate the point of threshold crossings. Both have a schematic inset of recording methods (left). **B.** Binarized overlayed behavior over time from the different parts of the video (top) and from the running band (bottom). Note the quantification in this animal of the proportion of synchronic run and whisking periods versus periods of whisking alone. **C.** Overlay of all extracted run bouts in all recordings (top); the pie plot indicates the proportion of active ("run") and passive ("rest") periods in the entire recording duration. Quantifications of its amplitude (bottom left) and duration properties on a logscale axis (bottom right). **D.** Dataset quantifications of time spent active ("All moves") versus rest (left); of the period with whisking and run versus whisking alone (middle left), of the periods related to different facial movements (middle right) and their respective correlations (right). Note that as expected, mouth, nose and whisking periods are strongly correlated.
